# A CCL4-enriched NK-cell transcriptional state is associated with inflammatory and myeloid-stromal programs in colorectal cancer liver metastasis

**DOI:** 10.3389/fimmu.2026.1923511

**Published:** 2026-09-16

**Authors:** Lihong Lin, Michael Zhengyang Xu, Xiaochun Gu, Weijian Zhang, Chun Feng, Wenlong Ming

**Affiliations:** 1 Jiangsu Key Laboratory of Intelligent Medical Image Computing, School of Artificial Intelligence, Nanjing University of Information Science and Technology, Nanjing, China; 2 Center of Interventional Radiology and Vascular Surgery, Department of Radiology, Zhongda Hospital, Medical School, Southeast University, Nanjing, China; 3 Department of Colorectal Surgery, Ningbo No. 2 Hospital, Ningbo, China; 4 Jinling High School Hexi Campus, Nanjing, China; 5 The Center of Joint and Sports Medicine, Orthopedics Department, Zhongda Hospital, School of Medicine, Southeast University, Nanjing, China

**Keywords:** cancer-associated fibroblasts, CCl4, colorectal cancer liver metastasis, natural killer cells, NF-κB, spatial transcriptomics, SPP1+ macrophages, tumor microenvironment

## Abstract

**Background:**

Colorectal cancer liver metastasis (CRLM) develops within a liver-specific immune and stromal microenvironment, yet the natural killer (NK)-cell adaptations associated with this metastatic niche remain incompletely defined.

**Methods:**

We integrated human single-cell RNA sequencing, an exploratory murine cross-species comparison, spatial transcriptomics, bulk transcriptomic cohorts, donor-level pseudobulk analyses, cell-cell communication, pseudotime and in silico perturbation analyses, together with multiplex immunofluorescence, conditioned-medium assays, ELISA and immunoblotting.

**Results:**

Across 73,486 cells from six anatomical sites, NK cells showed lower relative representation within the recovered T/NK compartment in primary tumors and liver metastases, while the residual compartment showed inflammatory remodeling. NK-cell reclustering identified CD56-associated and CD16-associated CCL4 source clusters that were analyzed jointly as a CCL4-enriched NK-cell transcriptional state characterized by NF-κB, TNF, MAPK and JAK-STAT programs. In paired GSE164522 samples from 10 patients, this state was independently recovered, with nominally higher CCL4-associated signal in liver metastases than in primary tumors that did not remain significant after Benjamini-Hochberg correction. Spatial and communication analyses associated the state with SPP1-positive macrophage and stromal programs without establishing causality. Tissue imaging, conditioned-medium assays, ELISA and immunoblotting provided complementary marker-level and model-system support for CCL4-associated inflammatory activity and NF-κB pathway responsiveness.

**Conclusion:**

CRLM is associated with altered NK-cell representation among recovered cells and a reproducible CCL4-enriched inflammatory NK-cell transcriptional state. Its association with inflammatory and myeloid-stromal programs across independent, spatial and model-system analyses provides a focused framework for future lineage-restricted and *in vivo* studies.

## Introduction

Colorectal cancer (CRC) remains a leading cause of cancer-related mortality worldwide, and liver metastasis is a major driver of treatment failure and poor outcome (1–3). CRLM lesions develop within a liver-specific microenvironment in which immune, myeloid, stromal and epithelial compartments are spatially organized rather than uniformly suppressed (4–6). Defining the cellular states that connect innate immune activation to these metastatic niches is therefore essential for understanding CRLM progression.

Natural killer (NK) cells are central components of innate anti-tumor immunity and can contribute to the elimination of circulating tumor cells and early metastatic deposits (7–11). In established tumors, however, NK cells undergo substantial state remodeling, including impaired cytotoxicity, altered trafficking, exhaustion-like features and transcriptional adaptation to stress signals in the tumor microenvironment (12–16). Current descriptions of tumor-infiltrating NK cells have therefore focused mainly on cytotoxic loss and exhaustion-like dysfunction (17). Less is known about whether NK cells in CRLM acquire tissue-specific inflammatory states and whether these states are linked to the architecture of the metastatic niche.

Chemokine-high NK states are of particular interest because chemokines can reflect immune activation while also shaping the recruitment and positioning of other cells in tumors. CCL4 is classically associated with immune-cell recruitment and immune contexture, but chemokine signaling can have divergent effects depending on tumor type, receptor context and cellular source (18, 19). In CRLM, macrophage-NK crosstalk and local chemokine gradients have been implicated in metastatic immune regulation (20, 21). These observations raise a key unresolved question: whether CCL4-expressing NK cells represent a bystander inflammatory signature, a host immune-engagement marker, or a state embedded within multicellular remodeling programs in metastatic tissue.

The second unresolved layer concerns the myeloid-stromal niche. Single-cell atlases have identified tumor-associated macrophage programs, including SPP1+ macrophages, as major components of immunoregulatory tumor ecosystems (22–29). In parallel, cancer-associated fibroblasts, pericytes and extracellular matrix programs can generate tissue barriers that shape tumor progression (30–34). Spatial profiling further indicates that these immune and stromal programs are organized into local multicellular hubs rather than dispersed randomly across tumor tissue (35–39). However, the innate inflammatory signals that link NK-cell remodeling to SPP1+ macrophage, myCAF and pericyte programs in CRLM remain insufficiently defined.

Here, we combined human and mouse CRLM single-cell transcriptomes with spatial transcriptomics, external bulk cohorts, cell–cell communication analysis, pseudotime modeling, in silico perturbation, target prioritization, multiplex immunofluorescence and *in vitro* assays to investigate NK-cell remodeling in CRLM (**Figure 1**). We focused on a CCL4^+^ NK-cell state enriched in CRLM lesions and its link to NF-κB activation and myeloid–stromal remodeling niches. This integrative analysis associated CCL4^+^ NK cells with an SPP1^+^ macrophage–myCAF/pericyte network and prioritized the IL1B/NF-κB/CCL4 program for future mechanistic investigation.

**Figure 1:**
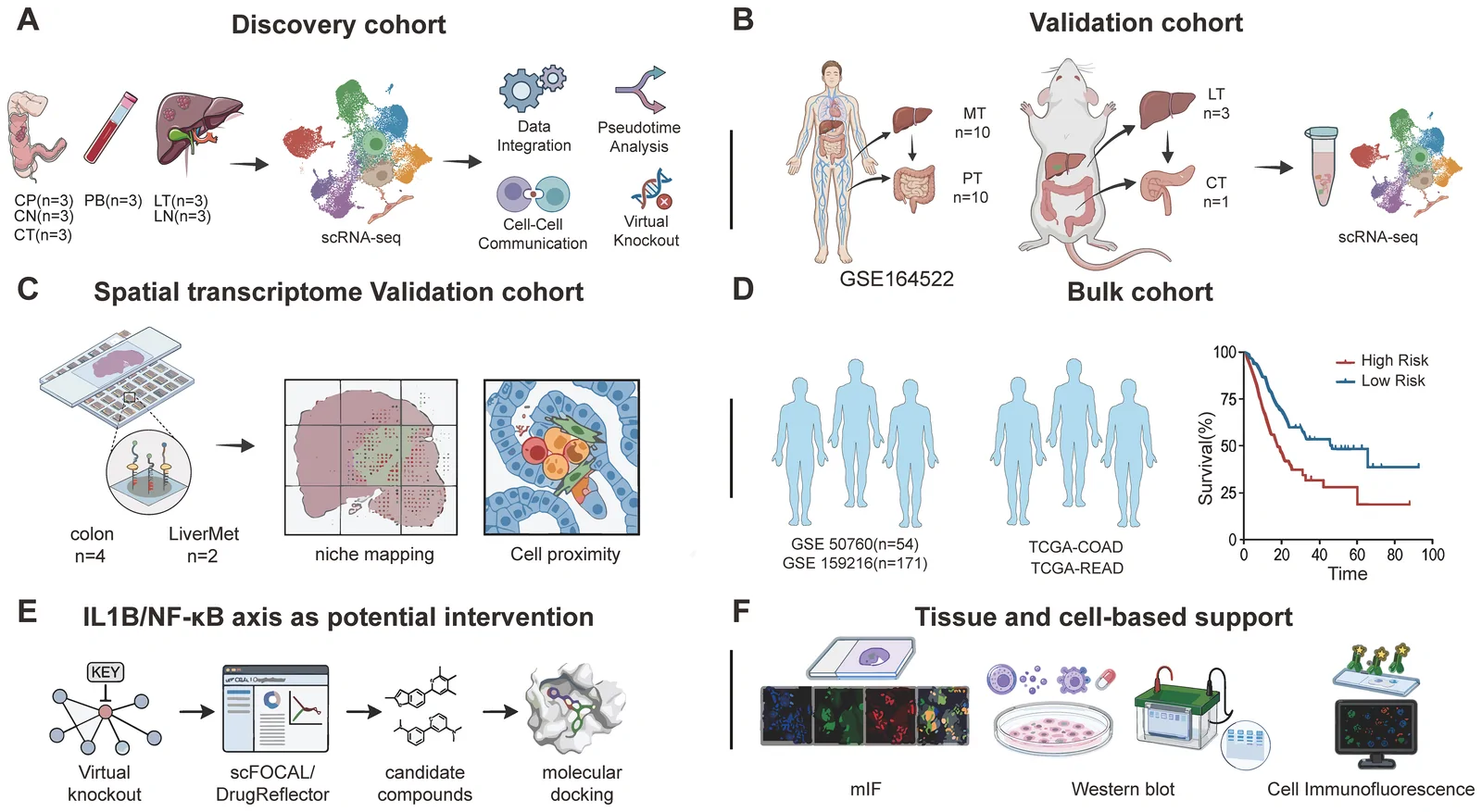
Study design and evidence hierarchy. **(A)** Discovery single-cell RNA sequencing (scRNA-seq) cohort assembled from Gene Expression Omnibus (GEO) datasets and comprising six tissue origins, including colon normal (CN), colorectal paratumor tissue (CP), primary colorectal tumor (CT), normal liver (LN), liver metastasis (LT), and peripheral blood (PB), with three samples per tissue group. Integrated scRNA-seq data were subjected to data integration, pseudotime analysis, cell–cell communication analysis, and virtual knockout analysis. **(B)** Independent supporting datasets comprising a publicly available paired colorectal cancer liver metastasis cohort (GSE164522), containing matched primary tumor and liver metastasis samples from 10 patients, and a separately generated in-house mouse single-cell RNA sequencing dataset comprising three liver metastasis samples and one colorectal tumor sample. **(C)** Spatial transcriptomics (ST) supporting cohort comprising four primary colorectal cancer (CRC) sections and two CRLM sections, analyzed by spatial niche mapping and cell-proximity analysis. **(D)** External bulk RNA sequencing (RNA-seq) cohorts used for transcriptomic and survival analyses, including GSE50760 (n = 54), GSE159216 (n = 171), The Cancer Genome Atlas Colon Adenocarcinoma (TCGA-COAD), and The Cancer Genome Atlas Rectum Adenocarcinoma (TCGA-READ). **(E)** Computational prioritization of the interleukin-1 beta (IL1B)/nuclear factor kappa B (NF-κB) axis as a candidate inflammatory program for mechanistic follow-up, integrating virtual knockout, Single-Cell Framework for Omics-Connectivity and Analysis via L1000 (scFOCAL), DrugReflector, candidate-compound prioritization, and molecular docking. **(F)** Tissue- and cell-based experimental support using multiplex immunofluorescence (mIF), Western blotting, and cellular immunofluorescence. Computational, spatial, mouse, and cell-model analyses are considered supportive or hypothesis-generating and are not presented as definitive causal or therapeutic validation.

## Materials and methods

### Study design and data sources

This study integrated single-cell RNA sequencing (scRNA-seq), spatial transcriptomics (ST), bulk RNA sequencing, TCGA cohorts, an independent paired human scRNA-seq cohort and mouse-model data to characterize immune and stromal remodeling in CRLM. The central analysis concerned a CCL4-enriched NK-cell transcriptional state and its associations with inflammatory, myeloid and stromal programs. Computational communication, trajectory, perturbation and docking outputs were treated as hypothesis-generating rather than confirmatory evidence.

Public scRNA-seq datasets GSE161277, GSE231559, and GSE245552 were obtained from the Gene Expression Omnibus (GEO) and integrated with the Sathe/Ji CRLM scRNA-seq dataset obtained from the Hanlee P. Ji Research Group data portal. Sample annotations were manually curated and assigned to six tissue origins: peripheral blood (PB), normal colon (CN), colorectal paratumor tissue (CP), primary colorectal tumor (CT), normal liver (LN) and liver metastasis (LT). The independent paired CRLM scRNA-seq cohort GSE164522, containing matched primary-tumor and liver-metastasis samples from 10 patients, was analyzed separately. Public spatial transcriptomics (ST) data (GSE225857) were used for spatial support. Bulk RNA-seq datasets included GSE50760, GSE159216, and GSE213402. Transcriptomic and clinical data for TCGA-COAD and TCGA-READ were downloaded from the Genomic Data Commons (GDC).

For GEO bulk cohorts, RNA-seq data were processed as raw counts, fragments per kilobase of transcript per million mapped reads (FPKM), or normalized expression matrices according to the source format. Microarray probes were mapped to gene symbols using the corresponding Gene Expression Omnibus Platform (GPL) annotation files. For genes represented by multiple probes, the mean probe value was used. TCGA samples were stratified into tumor and normal groups using sample barcodes and matched to available clinical follow up.

### Patient derived tumor xenograft mouse model

Patient derived tumor xenograft (PDTX) CRLM models were established for *in vivo* comparison (40). Fresh human tumor tissues were washed with phosphate buffered saline (PBS), minced with sterile surgical scissors and digested with 0.25% trypsin. Digestion was stopped by adding an equal volume of complete culture medium. The cell suspension was centrifuged at 1,000 r/min for 5 min, and the pellet was resuspended in PBS. Cell concentration was adjusted using an automated cell counter.

Twelve male BALB/c nude mice (7 weeks old) were housed in a temperature- and humidity-controlled environment (25 ± 1 °C, 70% humidity) with a 12-h light/dark cycle and ad libitum access to standard chow and sterile water. For modeling, mice were anesthetized, and an oblique ~1.0 cm incision was made below the intersection of the posterior axillary line and the costal margin to expose and gently exteriorise the lower pole of the spleen. Each mouse was injected with 0.1 ml of the cell suspension (~1 × 10^7^ cells/ml), equating to ~1 × 10^6^ tumor cells. A 1 ml sterile syringe was slowly advanced ~0.5 cm along the long axis of the spleen, and the suspension was injected beneath the splenic capsule over ~5 min. Following injection, hemostasis was achieved by compressing the puncture site with a 75% ethanol swab for 3–5 min. The spleen was returned to the abdominal cavity, and the abdominal wall was closed in layers. Splenectomy was not performed.

Postoperative activity, water intake, food consumption and general condition were monitored daily. In the early postoperative period, mice exhibited reduced activity, drinking and feeding, which recovered by ~5 days post-surgery. In the late postoperative period, mice developed progressive emaciation, reduced food intake, lethargy, sluggish movement and abdominal distension. Control mice were used to distinguish postoperative stress and non-neoplastic hepatic changes from baseline tissue morphology, assisting identification of tumor formation and metastasis-associated alterations. Mice were euthanized for tissue collection approximately 4–6 weeks post-modeling, or upon exhibiting signs of significant tumor burden (e.g., overt emaciation, abdominal distension or reduced activity), by cervical dislocation under deep isoflurane anesthesia (5% in 100% oxygen). The mouse comparison dataset comprised one CT sample and three LT samples.

### Single-cell suspension preparation, 10x library construction, and sequencing for mouse samples

Harvested mouse CT and LT tissues were immediately placed in pre-chilled PBS or tissue preservation buffer for single-cell suspension preparation under cold conditions. Tissues were mechanically minced and digested using a combination of collagenase IV, DNase I, and trypsin at 37 °C for 30–45 min, with intermittent pipetting to aid dissociation. The digested cell suspension was filtered through a 40-μm cell strainer to remove debris and clumps. Following centrifugation, resuspension, and red blood cell lysis, cell concentration and viability were evaluated using an automated cell counter. Samples with >80% viability proceeded to library construction.

Single-cell 3’ mRNA libraries were constructed using the 10x Genomics Chromium platform (41). Single-cell suspensions were encapsulated into Gel Bead-In-Emulsions (GEMs), followed by reverse transcription, cDNA amplification, fragmentation, adapter ligation and PCR enrichment. Quality-controlled libraries were sequenced on the Illumina NovaSeq 6000 platform. Raw sequencing data were processed using Cell Ranger v8.0.1 and aligned to the human-mouse combined reference genome (refdata-gex-GRCh38_and_GRCm39-2024-A) to distinguish human tumor cells from mouse-derived microenvironmental cells.

### Quality control, integration and cell type annotation of public scRNA-seq data

Public scRNA-seq data underwent sample-level quality control. Potential doublets were identified using DoubletFinder v2.0.3. For the human discovery cohort, the implemented mitochondrial-content threshold was percent.mt <25%; the <10% value reported previously was a documentation error. Cells with 500-5,000 detected genes and sample-specific nCount_RNA thresholds were retained. The previously labelled mitochondrial subset was renamed an undefined cluster because elevated mitochondrial transcripts alone did not establish a biological cell identity. Mitochondrial and ribosomal genes were excluded from downstream marker interpretation where specified.

Quality-controlled public data were normalized, subjected to variable-gene selection and integrated using Seurat v4.3.0 (42). Anchor-based integration was performed using the top 2,000 highly variable genes, followed by correction of sample and dataset batch effects using Harmony v1.2.3 (43). Principal component analysis (PCA), shared nearest-neighbor (SNN) graph construction and Louvain clustering (primary resolution = 0.5) were performed, with UMAP used for visualization. Cell-type annotation was determined based on canonical marker genes, differentially expressed genes identified using FindAllMarkers, the CellMarker 2.0 database (44) and published CRC/CRLM single-cell atlases.

### Quality control, integration and annotation of mouse scRNA-seq data

Mouse scRNA-seq data were processed independently from the public human datasets. Because the mouse samples contained both human tumor cells and mouse microenvironmental cells, independent filtering criteria were applied. Cells were retained if they met the following thresholds: nCount_RNA = 500–25,000, nFeature_RNA > 500, percent.mt < 50%, percent.rib < 60% and percent.hgb < 50%.

Quality-controlled data were processed using Seurat for normalization, variable-gene selection, dimensionality reduction, clustering and visualization. Human tumor cells were distinguished from mouse immune/stromal cells based on alignment to the combined reference genome, species origin and canonical marker genes. Major cell types and key subpopulations were subsequently annotated. For key cell states identified in the human single-cell atlas, human-to-mouse orthologous gene conversion was performed using msigdbr (45). The converted signatures were then scored in the mouse dataset to assess program-level overlap with the CCL4-enriched NK-cell state, SPP1-positive macrophages, neutrophils and myCAFs; these analyses were not interpreted as conservation of a stable lineage or *in vivo* mechanism.

### Cell state annotation and marker gene identification

Major annotated compartments included T/NK cells, myeloid cells, epithelial cells, fibroblast and stromal cells, endothelial cells, B cells and plasma cells. NK, myeloid, epithelial, fibroblast and stromal compartments were further subclustered at a primary resolution of 0.1. Marker genes for each cell state were identified using the FindAllMarkers function in Seurat with the Wilcoxon rank sum test. Filtering thresholds were set to min.pct = 0.25 and logfc.threshold = 0.25, with genes ranked by adjusted *P* value and average log2 fold change.

The CCL4-enriched NK-cell state was defined within the NK/NKT compartment by unsupervised subclustering, NK-lineage programs (including NKG7, KLRD1, TYROBP, FCER1G, GNLY, PRF1 and FCGR3A) and CCL4-associated multigene scores rather than binary CCL4 detection; for brevity, “CCL4+ NK cells” denotes this operationally defined state. The CD56-associated and CD16-associated source clusters were merged for primary downstream state-level analyses because they shared NK-lineage, CCL4/CCL5, NF-κB, TNF/MAPK/JAK-STAT and stress-response programs without robust evidence for distinct biological populations; selected source-cluster-resolved marker, pathway and functional-state analyses were retained to assess within-state heterogeneity and robustness of the shared program. T-lineage programs, including CD3D, CD3E, TRAC and TRBC, were reviewed to flag possible contamination. SPP1-positive macrophages were assigned using tumor-associated macrophage signatures, including SPP1, APOE, TREM2 and C1Q family genes. myCAFs, pericytes, neutrophils and stem-like epithelial cells were defined by canonical and cell-state-specific markers (23).

### Gene signature scoring and sample level pseudobulk aggregation

Cell state signatures, including CCL4+ NK cells, SPP1+ macrophages, neutrophils, myCAFs, pericytes and stem like epithelial cells, were constructed from subpopulation marker genes. Functional programs, including cytotoxicity, proinflammatory signaling, inhibitory/exhaustion, apoptosis and the NF-κB inflammatory program, were derived from marker genes and validated gene sets (46–48). At single-cell resolution, scores were calculated using the rank-based UCell method.

For the independent GSE164522 cohort, primary-tumor and liver-metastasis samples were paired within 10 patients and summarized at the patient level. Donor-level pseudobulk profiles were used for the reassessed differential-expression analyses, whereas the original cell-level **Figures 2F**, **3F** score results were not recomputed as donor-level inference.

**Figure 2:**
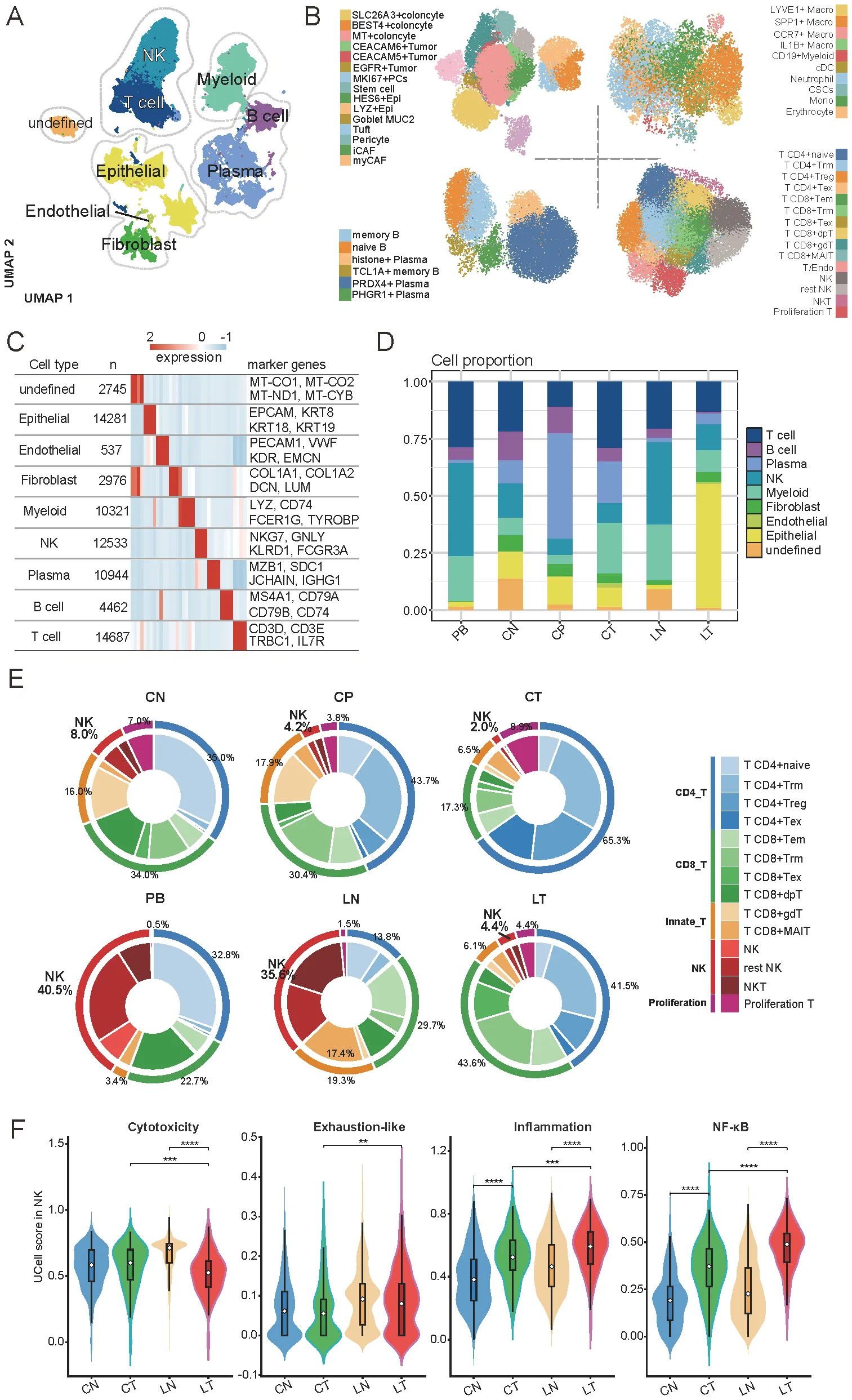
Cellular landscape across colorectal cancer liver metastasis-associated tissues. **(A)** UMAP of 73,486 quality controlled cells from the integrated human scRNA-seq cohort, showing major immune, stromal and epithelial compartments. **(B)** Reclustered UMAPs resolving epithelial/stromal, myeloid, B-cell/plasma-cell, and T-cell/NK-cell subpopulations. **(C)** Heatmap showing representative lineage-marker expression and cell numbers across the major cell compartments. NK-cell identity was supported by expression of NKG7, GNLY, KLRD1, and FCGR3A. **(D)** Relative abundance of major cell compartments across PB, CN, CP, CT, LN and LT samples (n=3 per tissue group). **(E)** Donut plots showing T/NK composition across tissue groups. The inner ring denotes broad T/NK classes and the outer ring denotes annotated T/NK states. **(F)** UCell score distributions for NK cell cytotoxicity, exhaustion like phenotype, inflammation and NF-κB programs in CN, CT, LN and LT tissues. Cell-level comparisons in this panel are descriptive and are not used for donor-level tissue-group inference.

**Figure 3:**
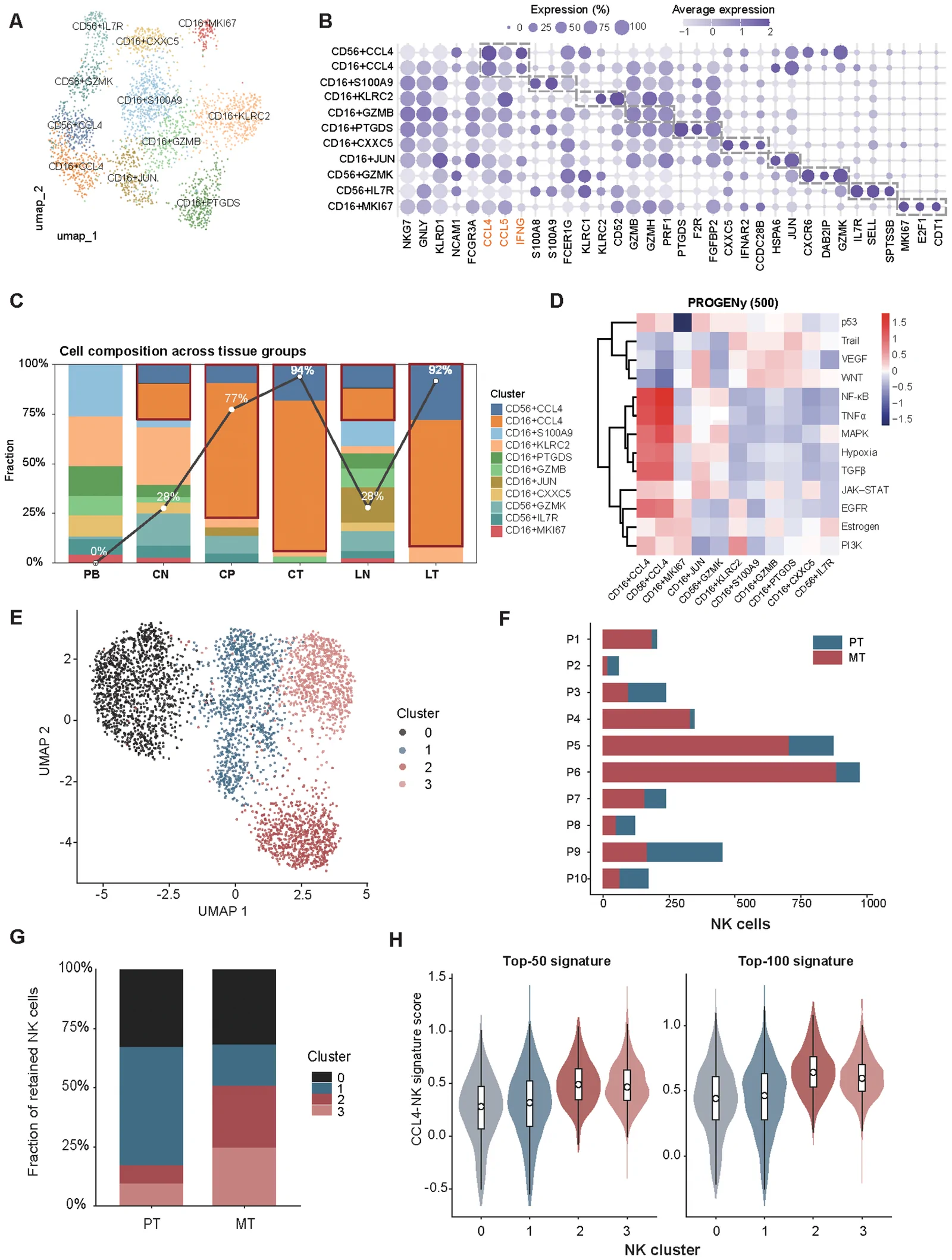
CCL4+ NK cells show an NF-κB associated inflammatory remodeling state. **(A)** Tissue-specific pathway remodeling in CCL4+ NK cells across PB, CN, CP, CT, LN, and LT, showing scaled pathway activities, including PI3K, hypoxia, p53, TNFα, and JAK–STAT signaling. **(B)** Inferred NK-cell pseudotime trajectory and associated transcriptional dynamics. Density plots show the distribution of NK states along pseudotime, and the heatmap shows three pseudotime-associated gene modules (C1–C3) together with representative enriched biological processes. **(C)** Functional module scores along pseudotime, including apoptosis, cytotoxicity, hypoxia/stress, inhibitory or exhaustion like programs, NF-κB and proinflammatory or chemokine programs. **(D)** Marker genes enriched in CCL4+ NK cells relative to other NK states, including FOS, JUND, NFKBIA and CCL4. **(E)** Hallmark NF-κB scores across NK states. **(F)** Hallmark NF-κB score distributions across tissue groups; cell-level comparisons are descriptive and are not used for donor-level tissue-group inference. **(G)** Differential transcription factor activity between LT and CT CCL4+ NK cells. **(H)** TF target network connecting candidate transcription factors with inflammatory target genes. Triangles denote transcription factors and circles denote target genes; node colour indicates scaled activity or expression change.

### PROGENy pathway activation and transcription factor activity inference

Signaling pathway activation at the single-cell or pseudobulk level was inferred using Pathway RespOnsive GENes (PROGENy) (49). PROGENy was implemented via decoupleR (50) using the human model and a target-gene weight network (top = 500). Analysed pathways included Androgen, EGFR, Estrogen, Hypoxia, JAK-STAT, MAPK, NF-κB, PI3K, TGF-β, TNF-α, TRAIL, VEGF, WNT and p53. For single-cell analysis, the RNA data matrix of CCL4-enriched NK cells was used as input, and scores were computed using decoupleR::run_mlm (minsize = 5).

Transcription factor (TF) activities were inferred using decoupleR coupled with CollecTRI (51). The regulatory network was retrieved using decoupleR::get_collectri (organism = “human”, split_complexes = FALSE), and TF activities were estimated via run_mlm. For donor-level differential expression, raw counts from total refined NK cells and the CCL4-enriched state were aggregated by donor and analyzed with edgeR quasi-likelihood models using TMM normalization, robust dispersion estimation and Benjamini-Hochberg correction. Leave-one-donor-out refits were used to assess directional sensitivity. Pathway and TF networks were visualised using Cytoscape v3.10.4 and interpreted as regulatory prioritization rather than direct causality.

### Pseudotime trajectory analysis, root inference, and identification of stage-specific gene patterns

Cell-state transition trajectories were inferred using Cytotrace2 v1.1.0, Slingshot and Monocle 3 v1.4.26 (52–54). Cytotrace2 aided evaluation of differentiation potential and relative initial states, Slingshot assisted trajectory directionality inference, and Monocle 3 was used to construct the principal graph, compute pseudotime and visualise trajectories.

Integrating these findings with tissue origins, cell-state distributions and early-progression marker expression, the CD56_IL7R state was designated as the root. For Monocle 3, RNA counts were extracted from the Seurat object to construct a cell_data_set. Preprocessing was performed using preprocess_cds(num_dim = 10), followed by batch-effect correction using Sample2 as the alignment_group. Dimensionality reduction, clustering, learn_graph and pseudotime calculations were then executed.

Pseudotime-associated genes were identified via graph_test, and the top 100 genes ranked by Moran’s I were selected for clustering and heatmap visualization. ClusterGVis was applied to perform k-means clustering on dynamically expressed genes (cluster.num = 3), identifying early-, mid-, and late-stage expression patterns. Pseudotime was segmented into phases to illustrate changes in cellular composition, pathway scores, and mechanistic genes.

### Differential expression and functional enrichment analysis

Differential expression analysis was conducted using the FindMarkers or FindAllMarkers functions in Seurat with the Wilcoxon rank-sum test. Thresholds were set to min.pct = 0.25 and logfc.threshold = 0.25, with an adjusted P value < 0.05 considered statistically significant. Functional annotation of differentially expressed genes was performed using clusterProfiler v4.6.2 for Gene Ontology (GO) and Kyoto Encyclopedia of Genes and Genomes (KEGG) enrichment analyses (55). Gene Set Enrichment Analysis (GSEA) used MSigDB C2 or Hallmark gene sets, with a significance threshold of false discovery rate (FDR) q < 0.05. Exploratory cell-level marker analyses were used for state characterization. Tissue-group differential expression was reassessed at donor level using edgeR pseudobulk profiles for total refined NK cells and the CCL4-enriched state.

### Cellular composition and cell state association analysis

Cellular composition was calculated within each biological sample using explicit denominators. Spearman’s rho was used to assess associations between the CCL4-enriched NK-cell state or signature and other cell programs, and Kendall’s tau was used for monotonic relationships between gene expression, signature scores, pathway activity and pseudotime. Correlation analyses used biological samples, patient-tissue samples or pseudotime-ordered states as statistical units, with multiple-testing correction where appropriate. These analyses describe co-variation among recovered cell states and do not establish absolute abundance, direct interaction, prognostic independence or causal remodeling.

### Cell–cell communication analysis

Cell-cell communication was analyzed using CellChat (56), NicheNet (57) and MultiNicheNet (58) to nominate candidate ligand-receptor relationships. Inferred probabilities represent expression-based signaling potential and do not demonstrate physical contact, signaling direction, necessity or sufficiency. The main text was restricted to prioritized CCL4-associated NK-myeloid relationships, including links to SPP1-positive macrophage programs; broader TGF-β, MIF, IL1, TNF, ICAM, SPP1, integrin and extracellular-matrix networks were retained as supplementary hypothesis-generating results.

NicheNet was applied to link candidate ligands to downstream target-gene programs, and MultiNicheNet was used to compare sample-level communication patterns between CT and LT conditions. Integrated marker, pathway, transcription-factor, communication and spatial evidence was used to nominate IL1B, PDGFRA, NAMPT, TGFB1 and CXCR4 as candidate regulatory nodes. These nodes were used to prioritize testable hypotheses and were not interpreted as direct targets or proof that a specific ligand-receptor edge is active.

### TCGA deconvolution, signature correlation, and survival analysis

Integrated bulk RNA-seq and clinical data from the TCGA-COAD and TCGA-READ cohorts were used for external support. Cell-state signatures were scored using single-sample gene set enrichment analysis/gene set variation analysis (ssGSEA/GSVA). Cellular composition was estimated via deconvolution using BayesPrism v2.2.2 with the integrated scRNA-seq atlas as a reference. Signature differences between tumor and normal tissues were evaluated using the Wilcoxon rank-sum test, and associations among signatures, deconvolution scores, and candidate ligand/receptor genes were assessed using Spearman correlation analysis. For overall survival, CCL4 expression and the CCL4-NK top-50 and top-100 signatures were Z-standardized and analyzed as continuous variables using Cox proportional-hazards models. Multivariable models adjusted for age, sex, AJCC stage, COAD/READ cohort and total NK-cell abundance; MSI was examined in a sensitivity analysis, and proportional-hazards assumptions were assessed using Schoenfeld residuals. Median-stratified Kaplan-Meier curves were retained only as exploratory visualizations.

### Cell-state signature scoring, spatial niche identification and colocalization analysis in ST

The public Visium ST dataset GSE225857 was used to assess the spatial distribution of key cell states. Cell-state signature scoring at the ST spot level was computed based on the top 50 highly ranked marker genes from the scRNA-seq analysis. For Giotto-based spatial niche analysis, four cell-state scores were calculated for each spot and incorporated into the Giotto object as spatial feature inputs. The resulting score matrix was scale-normalized across spots and clustered using k-means clustering, resulting in six spatial niches designated N0–N5. Mean scores were then calculated for each niche, and Z-score-normalized heatmaps were used to visualise the relative enrichment of CCL4+ NK, SPP1+ macrophage, myCAF and pericyte-associated spatial programs.

For spatial colocalization analysis, Spearman correlations among signature scores were calculated at the spot level. Signature scores were binarised to define high-scoring regions (75th percentile as the primary threshold; 70th percentile in select panels) to identify co-localized CCL4+ NK cell–SPP1+ macrophage high-scoring spots. Double-positive, triple-positive, and multi-state overlapping regions were defined via logical combinations and quantified using the Jaccard index and overlapping spot proportions. Composite spatial network scores were constructed by summing the respective normalized scores to summarise the degree of NK–myeloid–stromal spatial coupling.

### Spatial ligand-receptor pair scoring

To evaluate potential spatial cell–cell communication patterns, expression scores for candidate ligand–receptor pairs were calculated at the spot level (59). For single-ligand/single-receptor pairs, the score was calculated as the product of ligand and receptor expression values. For multi-subunit complexes, the expression of receptor-complex members was aggregated using the geometric mean before multiplication with the ligand expression value (60).

### scTenifoldKnk virtual knockout analysis

To assess computational network sensitivity to CCL4 within the CCL4-enriched NK-cell state, virtual knockout analysis was performed using scTenifoldKnk (61). RNA counts from the state were used as input. Lowly expressed genes, mitochondrial genes and ribosomal genes were excluded.

Running parameters were adjusted based on cell count: nc_nCells = min(500, max(150, floor(ncells × 0.7))), nc_nNet = 15, nc_nComp = 3 and n_runs = 2 (each with a distinct random seed). Significantly perturbed genes were filtered using thresholds of adjusted *P* value < 0.05 or |Z| ≥ 2.5 and ranked according to detection frequency, average Z-score, fold change and perturbation distance. Differentially regulated genes were subjected to GO/KEGG enrichment analyses and used for downstream drug prioritization.

### DrugReflector, scFOCAL, and drug prioritization

DrugReflector and scFOCAL were used as exploratory compound-prioritization tools. Disease/state-specific input signatures were constructed from directional gene sets, virtual-perturbation outputs and fixed cell-state signatures. Compound identities and ranking context were rechecked chemically. Ingenol mebutate was interpreted as a computationally nominated PKC/phorbol-ester-related perturbagen/probe.

For scFOCAL, the disease signature was computed in the overall mode, with a maximum of 500 tested cells per group. Reference perturbation states used IL7R- and GZMK-associated states as controls; candidate compound evaluation focused on ingenol mebutate. Grouping thresholds for sensitive and tolerant cells were set to -0.15 and 0.25, respectively, and differential expression analysis of the tolerance signature was conducted using MAST. PKC/phorbol-ester class enrichment and reverse-direction ranking were used to contextualize the perturbational signal; no therapeutic inference was made from rank alone.

### Molecular docking, redocking assessment, and GNINA rescoring

IL1B-related structures (monomer, IL1B-IL1R1 complex, IL1B-IL1RAcP complex and the 8C3U structure with co-crystallised ligand T9C) were retrieved from the Protein Data Bank (62). Receptor structures were prepared by removing water and non-target ligands, adding hydrogens and converting file formats. Ligands underwent conformation checking, hydrogen addition and energy minimisation before conversion to PDBQT format using Open Babel and Meeko (63, 64). Docking and rescoring were performed only as exploratory structure-based checks.

For redocking assessment, the co-crystallised ligand T9C in 8C3U was redocked, and docking parameter accuracy was assessed by calculating the heavy-atom root-mean-square deviation (RMSD) between the redocked and crystal poses. Ingenol and ingenol mebutate were subsequently docked into various *IL1B* contexts using AutoDock Vina, with the optimal affinity recorded and ranked based on the mean and standard deviation across repeated seeds (63, 65). The high-performing 1I1B *IL1B* monomer–ingenol mebutate system was subjected to GNINA rescoring, with a representative conformation selected based on GNINA affinity, CNNaffinity and CNNscore metrics (66). Results were visualised using PyMOL. Vina and GNINA scores served solely as computational predictors.

### Cell culture and conditioned medium preparation

SW620 human colorectal cancer cells, NK92 cells, THP-1 human monocytic cells and CCD-18Co human colon fibroblasts were used to establish an *in vitro* tumor–NK cell–macrophage–fibroblast cascade. SW620 cells were cultured in DMEM supplemented with 10% fetal bovine serum. NK92 cells were maintained in NK92 complete medium supplemented with interleukin-2. THP-1 human monocytic leukemia cells were cultured in RPMI-1640 medium supplemented with 10% fetal bovine serum and 0.05 mM 2-mercaptoethanol. THP-1 cells were induced toward macrophage-like cells by PMA treatment before conditioned-medium stimulation. CCD-18Co cells were cultured in MEM supplemented with 10% fetal bovine serum, 1% non-essential amino acids and 1 mM sodium pyruvate, and were used as a fibroblast model to evaluate α-SMA-associated myofibroblastic activation. All cells were maintained at 37 °C in a humidified incubator containing 5% CO_2_ and were routinely monitored for mycoplasma contamination.

To prepare tumor-conditioned medium, SW620 cells were grown to approximately 70-80% confluence, washed with PBS and incubated in serum-reduced medium containing 1% fetal bovine serum for 24 h. NK92 cells were treated for 24 h with SW620-conditioned medium mixed 1:1 with NK92 complete medium, and the resulting supernatant was collected as tumor-conditioned NK-conditioned medium. To address tumor-medium carryover, a matched direct Tumor-CM condition applied SW620-conditioned medium to macrophages without prior NK92 exposure; medium proportion, treatment volume and macrophage exposure time were matched to the Tumor-NK CM condition. THP-1-derived macrophages were exposed to the indicated conditioned media for 24 h, with CCL4-neutralizing antibody added where specified. For fibroblast-response assays, CCD-18Co cells were treated for 48 h with macrophage-conditioned medium collected from the indicated upstream macrophage cultures. All *in vitro* experiments were performed with three independent biological replicates, and the independent experiment was the inferential unit.

### Immunoblotting

NK92 cells subjected to the indicated treatments were harvested and washed twice with cold PBS. Total proteins were extracted using a total protein extraction reagent supplemented with phosphatase inhibitor, protease inhibitor and PMSF. Cell lysates were incubated on ice or at 4 °C with gentle agitation and then centrifuged at 14,000 rpm for 15 min at 4 °C. The supernatants were collected as total protein lysates, and protein concentrations were determined using a BCA protein assay. Equal amounts of protein were mixed with 5× SDS-PAGE loading buffer, denatured, separated by SDS-PAGE and transferred onto NC membranes using a Trans-Blot Turbo transfer system.

After transfer, membranes were washed with TBST and blocked with 5% non-fat milk for 2 h at room temperature. The membranes were then incubated overnight at 4 °C with primary antibodies against phosphorylated p65 (p-p65; Affinity, AF2006; 1:1000), total p65 (Affinity, AF5006; 1:1000), CCL4 (Affinity, DF6545; 1:1000), cleaved caspase-3 (Affinity, AF7022; 1:1000) and GAPDH (KeyGEN BioTECH, KGC6102-1; 1:500). After washing with TBST, membranes were incubated with HRP-conjugated goat anti-rabbit IgG secondary antibody (KeyGEN BioTECH, KGC6202-0.1) for 2 h at room temperature. Protein bands were visualised using an enhanced chemiluminescence detection system and imaged with a Bio-Rad ChemiDoc MP imaging system.

Band intensities were quantified using Gel-Pro32 software. p-p65 levels were normalized to total p65, whereas CCL4 and cleaved caspase-3 levels were normalized to GAPDH. For each independent experiment, normalized values were further expressed relative to the control group. Quantitative data were generated from three independent experiments and are presented as mean ± SEM. Ingenol mebutate perturbation was interpreted as PKC/NF-κB/CCL4 program responsiveness rather than therapeutic reversal or IL1B target engagement.

### Cellular immunofluorescence

Cellular immunofluorescence was performed to assess CCL4-associated signal in NK92 cells, IL-1β expression in THP-1-derived macrophages and α-SMA-associated fibroblast activation in CCD-18Co cells. After the indicated treatments, cells were fixed with 4% paraformaldehyde, permeabilized when intracellular staining was required and blocked with bovine serum albumin. Cells were incubated with primary antibodies against CCL4, IL-1β or α-SMA, followed by fluorophore-conjugated secondary antibodies and DAPI counterstaining. Secreted CCL4/MIP-1β in paired NK92 control and tumor-CM-stimulated supernatants was quantified by ELISA across three biological replicates.

Fluorescence images were acquired using an Olympus CKX53 fluorescence microscope. Within each staining panel, all treatment groups were imaged using identical exposure times and acquisition settings to ensure comparability. Image-based quantification used three independent biological experiments per condition; cells within images were used for segmentation and distributional visualization, but the independent experiment was the statistical unit.

### Multiplex immunofluorescence

Multiplex immunofluorescence was performed on FFPE human primary colorectal tumor and liver metastatic tissue sections. Sections of 4 μm thickness were deparaffinized, rehydrated and subjected to heat-induced antigen retrieval. Sequential staining was performed using a tyramide signal amplification-based multiplex fluorescence protocol.

For assessment of the inflammatory phenotype of the CCL4-enriched NK-cell state, archived primary colorectal tumor and liver-metastasis sections were stained with antibodies against NKp46/NCR1, CCL4, p-p65 and IL-1β, followed by DAPI counterstaining. Twelve spatially dispersed high-resolution ROIs were selected from each tissue without selecting only high-signal regions; one LT ROI failed the prespecified nucleus-count quality-control threshold. For liver-metastasis myeloid/stromal context, a separate panel stained DAPI, NKp46, CCL4, CD68 and α-SMA. The following primary antibodies were used where applicable: anti-NKp46/NCR1 (Abcam, ab324692, 1:500), anti-CCL4/MIP-1β (Abcam, ab308044, 1:300), anti-p-p65/NF-κB (Abcam, ab28856, 1:1000), anti-IL-1β (Proteintech/Sanying, 26048-1-AP, 1:2000), anti-CD68 (Cell Signaling Technology, 76437, 1:3000) and anti-α-SMA (Cell Signaling Technology, 19245T, 1:2000).

Whole-tissue sections were scanned using a Pannoramic MIDI II digital pathology slide scanner. Single-channel images were reviewed before merging to assess staining specificity. NKp46/NCR1 and CCL4 fluorescence intensities were analyzed as continuous signals because no validated binary positivity threshold was available. The NF-κB mIF panel yielded 23 QC-passed ROIs and 13,585 segmented nuclei for quantitative marker-level analysis. The liver-metastasis NKp46/CCL4/CD68/α-SMA panel yielded 65,945 segmented cells for descriptive nearest-neighbor analysis. These archived-tissue measurements provide marker-level spatial support but not CD45/CD3 lineage-restricted validation, direct cell-cell contact, signaling direction or causality.

### Statistical analysis

Statistical analyses were performed in R v4.5.3 and Python 3.10. Unless otherwise specified, tests were two-sided and the biological sample or patient was the inferential unit. Paired tests were used only for genuinely paired samples; the discovery CT and LT donors were treated as unpaired groups. Multiple comparisons were controlled with the Benjamini-Hochberg method where applicable. Cell-level analyses were descriptive unless explicitly stated, and nominal P values were distinguished from adjusted P values. Cox models reported hazard ratios, 95% confidence intervals and proportional-hazards checks.

## Results

### Lower relative NK-cell representation with inflammatory features in CRLM

To define the cellular composition of NK-cell remodeling in colorectal cancer liver metastasis (CRLM), we analyzed an integrated single-cell transcriptomic dataset spanning PB, CN, CP, CT, LN, and LT. After quality-control filtering, 73,486 cells were retained across 18 samples, with expected distributions of cell recovery, detected genes, transcript counts and mitochondrial content (**Supplementary Figures 1A–C**). Uniform manifold approximation and projection (UMAP) resolved the major immune, stromal and epithelial compartments, including T cells, NK cells, B cells, plasma cells, myeloid cells, fibroblasts, endothelial cells and epithelial cells (**Figure 2A**). Major cell populations were annotated according to canonical marker gene expression. Epithelial cells expressed EPCAM, KRT8, KRT18 and KRT19; endothelial cells expressed PECAM1, VWF, KDR and EMCN; fibroblasts expressed COL1A1, COL1A2, DCN and LUM; myeloid cells expressed LYZ, CD74, FCER1G and TYROBP; NK cells expressed NKG7, GNLY, KLRD1 and FCGR3A; plasma cells expressed MZB1, SDC1, JCHAIN and IGHG1; B cells expressed MS4A1, CD79A, CD79B and CD74; and T cells expressed CD3D, CD3E, TRBC1 and IL7R. Cells with high expression of mitochondrial genes, including MT-CO1, MT-CO2, MT-ND1 and MT-CYB, were annotated as undefined clusters (**Figures 2B, C**, **Supplementary Figures 1D**, **2A-F**).

Cellular composition differed substantially across anatomical compartments. At the major-lineage level, tissue samples showed distinct epithelial, stromal, myeloid and lymphoid representation, whereas PB and LN were dominated by immune compartments (**Figure 2D**). Within the T/NK compartment, NK cells accounted for 40.5% of cells in PB and 35.6% in LN, but only 8.0% in CN, 4.2% in CP, 2.0% in CT and 4.4% in LT (**Figure 2E**). Thus, NK cells had lower relative representation within the recovered T/NK compartment in CT and LT than in PB and LN.

Beyond this compositional shift, the residual NK compartment also showed transcriptional remodeling. Using curated gene sets for cytotoxic, exhaustion-like, inflammatory and NF-κB-associated programs (**Supplementary Table 1**), we scored NK cells from CN, CT, LN and LT tissues. At the cell level, LT-derived NK cells showed lower cytotoxicity scores than NK cells from LN, whereas inflammatory and NF-κB program scores were higher in tumor-associated and metastatic tissues (**Figure 2F**). These score distributions are descriptive and are not used as donor-level tissue-group inference. Together, the recovered-cell composition and functional-score patterns motivated state-level analysis of inflammatory and NF-κB-associated programs.

### CCL4+ NK cells show a pro-inflammatory state within the tumor microenvironment

Because NK cells showed tissue-dependent functional remodeling, we subclustered the NK compartment to resolve the cell states underlying these changes. Subclustering of 12,533 NK cells identified 11 NK-cell states, including CD56+ IL7R, CD56+ GZMK, CD56+ CCL4, CD16+ CCL4, CD16+ S100A9, CD16+ KLRC2, CD16+ GZMB, CD16+ PTGDS, CD16+ JUN, CD16+ CXCR5 and CD16+ MKI67 clusters (**Figure 4A**). Among these states, two clusters showed prominent CCL4 expression and were annotated as CD56+ CCL4 and CD16+ CCL4 NK cells.

**Figure 4:**
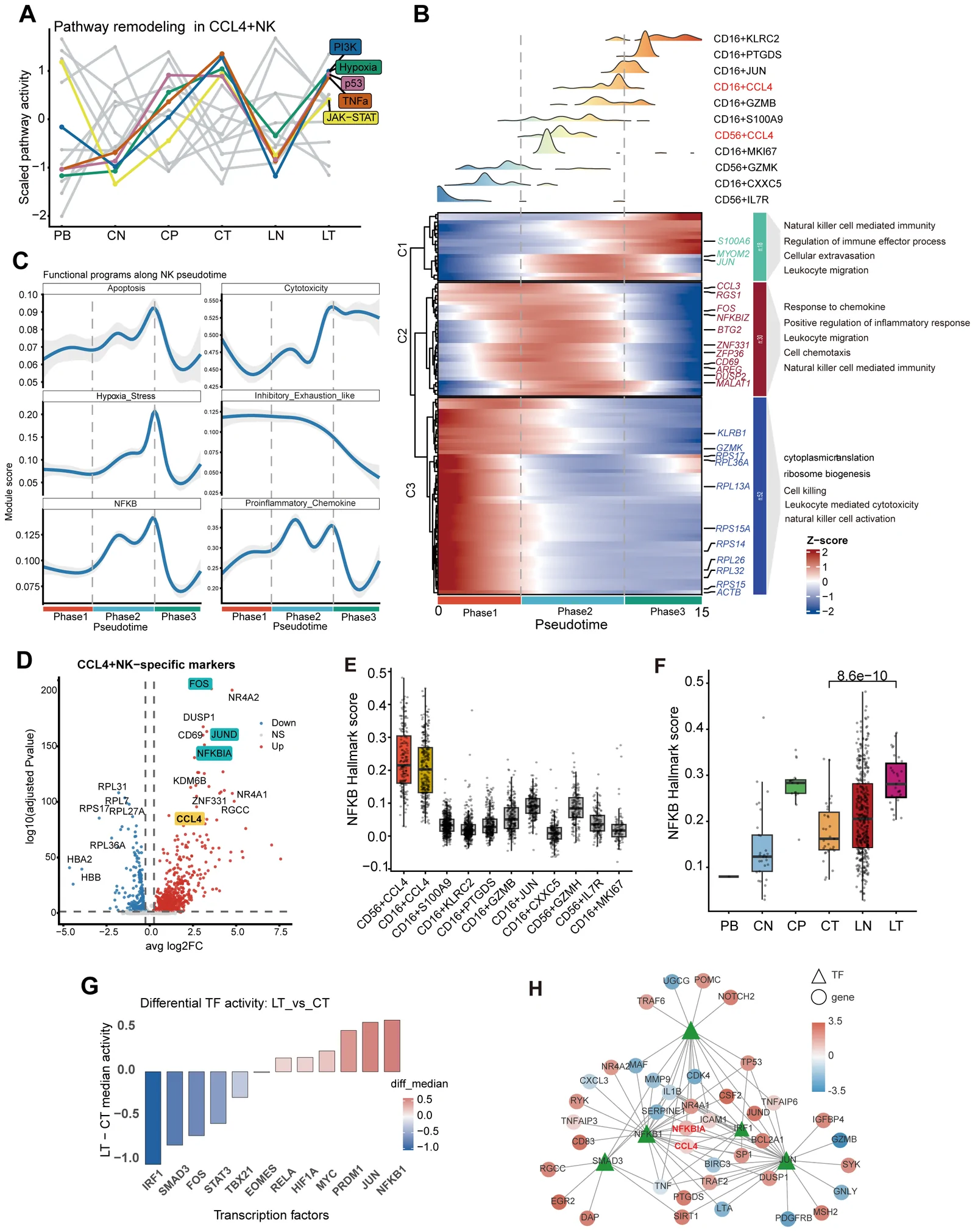
Tumor and metastasis-enriched CCL4+ NK states show inflammatory pathway activation and independent paired-cohort reproducibility. **(A)** UMAP of 12,533 reclustered NK cells, identifying CD56+ CCL4 and CD16+ CCL4 states among CD56+ and CD16+ NK populations. **(B)** Dot plot of representative NK-cell and inflammatory marker genes. Dot size denotes the percentage of expressing cells and color denotes scaled mean expression. **(C)** Compartment-specific distribution of NK states across PB, CN, CP, CT, LN and LT samples. The line denotes the combined fraction of CD56+ CCL4 and CD16+ CCL4 NK cells. **(D)** PROGENy-inferred pathway activation across NK states, showing increased NF-κB, TNF, MAPK, hypoxia, TGFβ and JAK-STAT signaling in CCL4+ NK states. **(E)** GSE164522 NK-cell reclustering after extraction and lineage filtering, showing the corresponding CCL4-associated populations. **(F)** Sample-level recovery and paired primary-tumor/liver-metastasis composition across 10 patients. **(G)** Aggregate distribution of NK-cell clusters 0–3 in PT and MT samples from the paired cohort. **(H)** Concordant top-50 and top-100 multigene score recovery in the paired cohort. State assignment combines NK-lineage programs, unsupervised clustering and multigene scores rather than binary CCL4 detection.

The two CCL4+ NK states retained canonical features of NK-cells while expressing genes associated with inflammatory and chemokines. Both states expressed core NK markers, including NKG7, GNLY, KLRD1, NCAM1 and FCGR3A, together with CCL4, CCL5 and IFNG (**Figure 4B**). Differential marker analysis further identified an immediate-early and inflammatory regulatory program in CCL4+ NK cells, including FOS, NR4A2, DUSP1, JUND, CD69, NFKBIA, JUNB, DUSP2, FOSB, ZFP36 and TNFAIP3 (**Supplementary Table 2**). These data are consistent with an operational NK-lineage transcriptional state characterized by inflammatory activation, while stress-response overlap remains a limitation.

The distribution of NK-cell states showed clear tissue-specific differences. The combined percentage of CD56+ and CD16+ CCL4+ NK cells was minimal in PB, CN and LN, but was markedly enriched in tumor-associated tissues, accounting for 77% of NK cells in CP, 94% in CT and 92% in LT (**Figure 4C**). This distribution indicates that CCL4+ NK cells are enriched within the primary colorectal tumor and liver metastatic microenvironments.

Pathway activity analysis supported the inflammatory identity of these tumor- and metastasis-enriched NK states. PROGENy inference showed that CD56+ CCL4 and CD16+ CCL4 NK cells had increased NF-κB, TNF, MAPK, hypoxia, TGFβ, JAK-STAT and EGFR pathway activities compared with other NK subsets (**Figure 4D**). Additional differential-expression and enrichment analyses linked CCL4+ NK-cell markers to TNF/NF-κB signaling, apoptosis-related and stress-associated programs (**Supplementary Figures 3A, B**). At the bulk-tissue level, CCL4 expression was higher in metastatic cancer than in primary colorectal cancer and normal colon in GSE50760, and CCL4+ NK marker genes were enriched among liver-metastasis-upregulated genes in GSE213402 (**Supplementary Figures 5A–D, F**, **Supplementary Table 3**). The CCL4+ NK ssGSEA score also correlated with the NF-κB ssGSEA score in GSE50760 (R = 0.84, p < 2.2e-16; **Supplementary Figure 5E**). Together, these discovery and external-cohort analyses supported classification of CCL4+ NK cells as a tumor-associated NK state characterized by inflammatory and NF-κB signaling. Continuous Cox analyses in TCGA-COAD/READ did not identify a stable independent survival association for CCL4 expression or the CCL4-NK top-50/top-100 signatures after available clinical and total-NK adjustment; complete model outputs are provided in the **Supplementary Materials** (**Supplementary Figure 5G**).

To test whether the CCL4-associated state could be reproduced in an independent CRLM cohort, we separately processed GSE164522 by extracting high-confidence NK cells from matched primary-tumor (PT) and liver-metastasis (MT) samples from 10 patients. After NK-lineage filtering and review of T-lineage contamination, unsupervised reclustering identified CCL4-associated source clusters 2 and 3, which retained NK-lineage markers together with CCL4-associated inflammatory programs (**Figure 4E**). Downstream comparisons were summarized at the patient, patient-condition, or sample level rather than treating individual cells as independent biological replicates.

At the paired patient level, the CCL4 top-100 score was nominally higher in MT than in PT (paired Wilcoxon P = 0.037; BH = 0.130), and the fraction of recovered NK cells with detectable CCL4 transcript was also nominally higher in MT (P = 0.014; BH = 0.096; **Supplementary Figure 4A**). These two measurements support increased CCL4-associated signal in MT in the paired cohort, while the BH-adjusted values indicate that they should be interpreted as supportive trends rather than definitive multiple-testing-adjusted tissue enrichment.

We then asked whether the CCL4-associated signal was linked to functional programs in the independent cohort. Across 20 patient-condition pseudobulk samples, the CCL4 top-100 score correlated with proinflammatory (Spearman rho = 0.729, P = 3.92 x 10^-4) and TNF/NF-κB scores (rho = 0.883, P < 2.2 x 10^-16; **Figures 4F–H**, **Supplementary Figure 4B**). In sample-level paired comparisons, CCL4-associated clusters 2/3 showed lower cytotoxic scores than clusters 0/1 (P = 9.57 x 10^-5; BH = 2.60 x 10^-4) and higher TNF/NF-κB scores (P = 0.018; BH = 0.031), whereas proinflammatory scores showed a non-significant trend (P = 0.089; BH = 0.125) and inhibitory/exhaustion-like scores did not differ (P = 0.751; BH = 0.751; **Supplementary Figure 4C**). Together, these analyses support independent recovery of a CCL4-enriched NK transcriptional state coupled to inflammatory/TNF-NF-κB programs, without implying single-cell-level independence or causal signaling.

### CCL4+ NK cells show an NF-κB-linked inflammatory state

The enrichment of CCL4+ NK cells reflected a tissue-restricted inflammatory state rather than a chemokine-high profile alone. Within the CCL4+ NK population, pathway activation scores showed substantial tissue-specific heterogeneity. Specifically, tumor-associated and metastatic samples displayed coordinated upregulation of PI3K, hypoxia, p53, TNF, and JAK-STAT pathways, alongside related stress and inflammatory signaling (**Figure 3A**). These findings show that CCL4+ NK cells exhibit tissue-associated transcriptional variation across distinct microenvironments of the CRLM ecosystem.

Trajectory analysis positioned the CCL4+ NK states along an inferred NK-cell remodeling trajectory. In pseudotime, CD56+ CCL4 and CD16+ CCL4 cells occupied intermediate-to-late regions of the trajectory. The associated dynamic gene modules were enriched for chemokine response, inflammatory response, leukocyte migration and NK cell-mediated immunity (**Figure 3B**, **Supplementary Figures 3E, H**). Functional module scoring along pseudotime showed that proinflammatory/chemokine, hypoxia/stress, NF-κB and apoptosis programs rose during the remodeling trajectory, whereas inhibitory/exhaustion-like scores declined and cytotoxicity changed dynamically across later states (**Figure 3C**, **Supplementary Figures 3F, G**). These patterns were consistent with an inferred continuum associated with inflammatory activation, stress response and NK effector remodeling.

State-specific marker analysis further supported the inflammatory regulatory identity of CCL4+ NK cells. Compared with other NK states, CCL4+ NK cells upregulated immediate-early and NF-κB-associated genes, including FOS, NR4A2, DUSP1, JUND, CD69, NFKBIA, CCL4, NR4A1 and TNFAIP3 (**Figure 3D**, **Supplementary Table 2**). Consistently, LT versus CT differential-expression analysis showed increased inflammatory regulators in CCL4+ NK cells, including JUND, while cytotoxic granule genes such as GZMB and GZMH were lower in LT-associated NK cells (**Supplementary Figure 3A**). NF-κB PROGENy-weighted genes, including NFKBIA, TNFAIP3, CCL4, ICAM1, CSF2 and CD83, were also shifted towards higher expression in LT relative to CT (**Supplementary Figure 3C**).

We then linked this inflammatory state to NF-κB activity. Hallmark NF-κB scores were among the highest in the CD56+ CCL4 and CD16+ CCL4 clusters compared with other NK subclusters (**Figure 3E**). Across tissue sites, cell-level NF-κB score distributions were higher in LT than in LN (**Figure 3F**); this panel is descriptive and is not used for donor-level tissue-group inference. Transcription factor activity analysis further identified differential activity between LT and CT in regulators including NFKB1, JUN, PRDM1, MYC, HIF1A, RELA, STAT3, FOS, SMAD3 and IRF1 (**Figure 3G**). A TF-target network connected NFKB1, JUN, RELA and STAT3 with inflammatory and chemokine-associated targets, including CCL4, NFKBIA, IL1B, CSF2, ICAM1 and TNFAIP3 (**Figure 3H**). These trajectory, marker, pathway and regulatory analyses were consistent with CCL4+ NK cells representing an NF-κB-linked inflammatory remodeling state in CRLM, while remaining consistent with an inferred regulatory association rather than direct transcription-factor causality.

### Tumor-conditioned cues increase CCL4 expression in NK cells

To examine whether the computationally defined CCL4+ NK inflammatory state was detectable at the tissue level, we performed multiplex immunofluorescence (mIF) in human colorectal tumor and liver metastatic tissue sections. In primary colorectal tumor tissue, representative regions contained NKp46/NCR1 and CCL4 protein signals, together with p-p65/NF-κB and IL-1β signals in the surrounding inflammatory tissue context (**Figure 5A**, **Supplementary Figure 4D**). Liver metastatic tissue contained NKp46/NCR1+ CCL4+ regions, with p-p65/NF-κB and IL-1β staining in adjacent tumor and stromal areas (**Figure 5B**, **Supplementary Figure 4D**). These representative tissue-level observations were consistent with the single-cell inference that CCL4+ NK cells occur within an NF-κB-linked inflammatory microenvironment. In descriptive marker-level analyses, NKp46/NCR1 and CCL4 fluorescence intensities were positively associated at the cell level in both primary tumor (Spearman rho = 0.46) and liver metastasis (rho = 0.27), with concordant ROI-median correlations (rho = 0.51 and 0.34, respectively).

**Figure 5:**
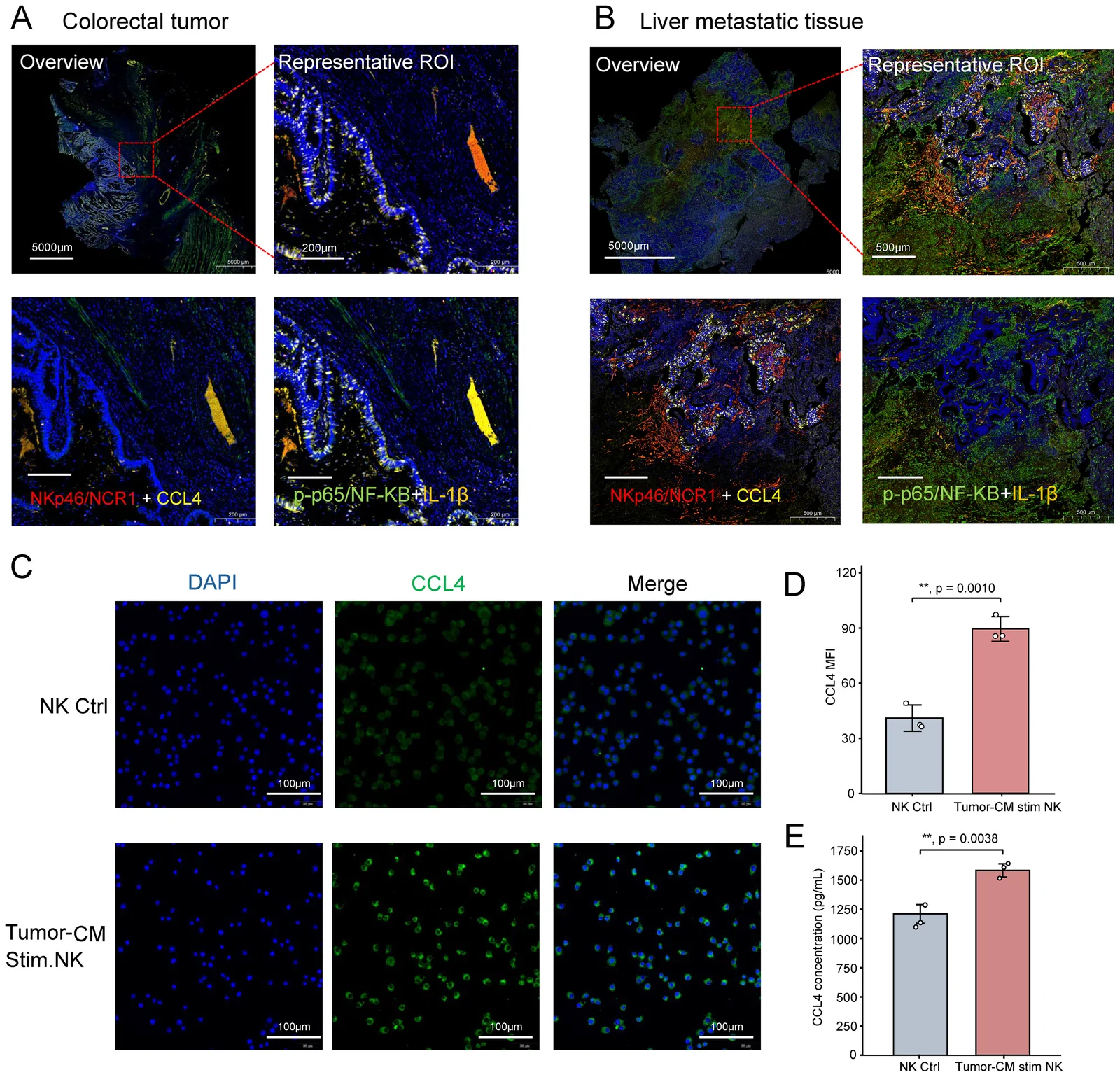
Tissue- and cell-based assays provide experimental evidence for increased CCL4 expression in NK cells within an inflammatory NF-κB and IL-1β context. **(A)** Multiplex immunofluorescence of human colorectal tumor tissue showing NKp46/NCR1, CCL4, p-p65/NF-κB and IL-1β staining in representative regions. **(B)** Multiplex immunofluorescence of human liver metastatic tissue showing representative NKp46/NCR1+ CCL4+ regions with adjacent p-p65/NF-κB and IL-1β signal. Overview and representative region-of-interest images are shown; scale bars are indicated in the panels. **(C)** Cellular immunofluorescence showing CCL4 signal in control NK92 cells and tumor conditioned medium (Tumor-CM) stimulated NK92 cells. Nuclei were counterstained with DAPI. Scale bars, 100 μm. **(D)** Quantification of CCL4 mean fluorescence intensity (MFI) in control and Tumor-CM stimulated NK92 cells. Data are presented as mean ± SEM from three independent biological replicates; exact P value is reported in the panel. **(E)** ELISA quantification of secreted CCL4/MIP-1β in three paired NK92 biological replicates after control or tumor-conditioned-medium stimulation; values are mean ± SD and the paired t-test P value is reported in the panel.

We then asked whether soluble tumor-derived factors induce CCL4 expression in NK cells *in vitro*. NK92 cells cultured in supernatant from colorectal cancer cells (Tumor CM) exhibited stronger CCL4 immunofluorescence than control cells (**Figure 5C**). Quantification of CCL4 mean fluorescence intensity confirmed a significant increase after Tumor-CM stimulation (**Figure 5D**). Secreted CCL4/MIP-1β levels were also significantly increased after Tumor-CM stimulation across three paired biological replicates (**Figure 5E**; paired t-test, P = 0.0038). These data support tumor-derived soluble-factor induction of CCL4 protein output in the NK92 model.

Together, the tissue and cell-model data support the presence of CCL4+ NK-cell signals in an IL-1β/NF-κB-rich inflammatory context. The added ELISA measurement further supports tumor-conditioned induction of CCL4 secretion in NK92 cells, while these model-system observations do not establish the origin or *in vivo* function of all CCL4+ NK cells.

### CCL4+ NK cells are linked to SPP1+ macrophages and stromal cells

Having characterized CCL4+ NK cells as an inflammatory NK-cell state, we next related this state to broader multicellular remodeling in CRLM. Across samples, the CCL4+ NK-cell proportion correlated positively with myeloid and fibroblast compartments and negatively with epithelial cells (**Figure 6A**, **Supplementary Figures 6A–E**). Correlation analysis among major cell compartments further showed coordinated variation between NK, myeloid and stromal lineages (**Figure 6B**, **Supplementary Figure 7A**). At finer resolution of parenchymal and stromal subsets, CCL4+ NK abundance correlated most strongly with myCAFs (rho = 0.65), stem-like epithelial cells (rho = 0.56) and pericytes (rho = 0.49). An EGFR+ tumor epithelial subset showed a negative correlation (**Figure 6C**, **Supplementary Figures 6B**, **7B, C**). Bootstrap analysis after normalization within the NK compartment supported the stability of the positive correlations with myCAFs, stem-like epithelial cells and pericytes (**Figure 6D**).

**Figure 6:**
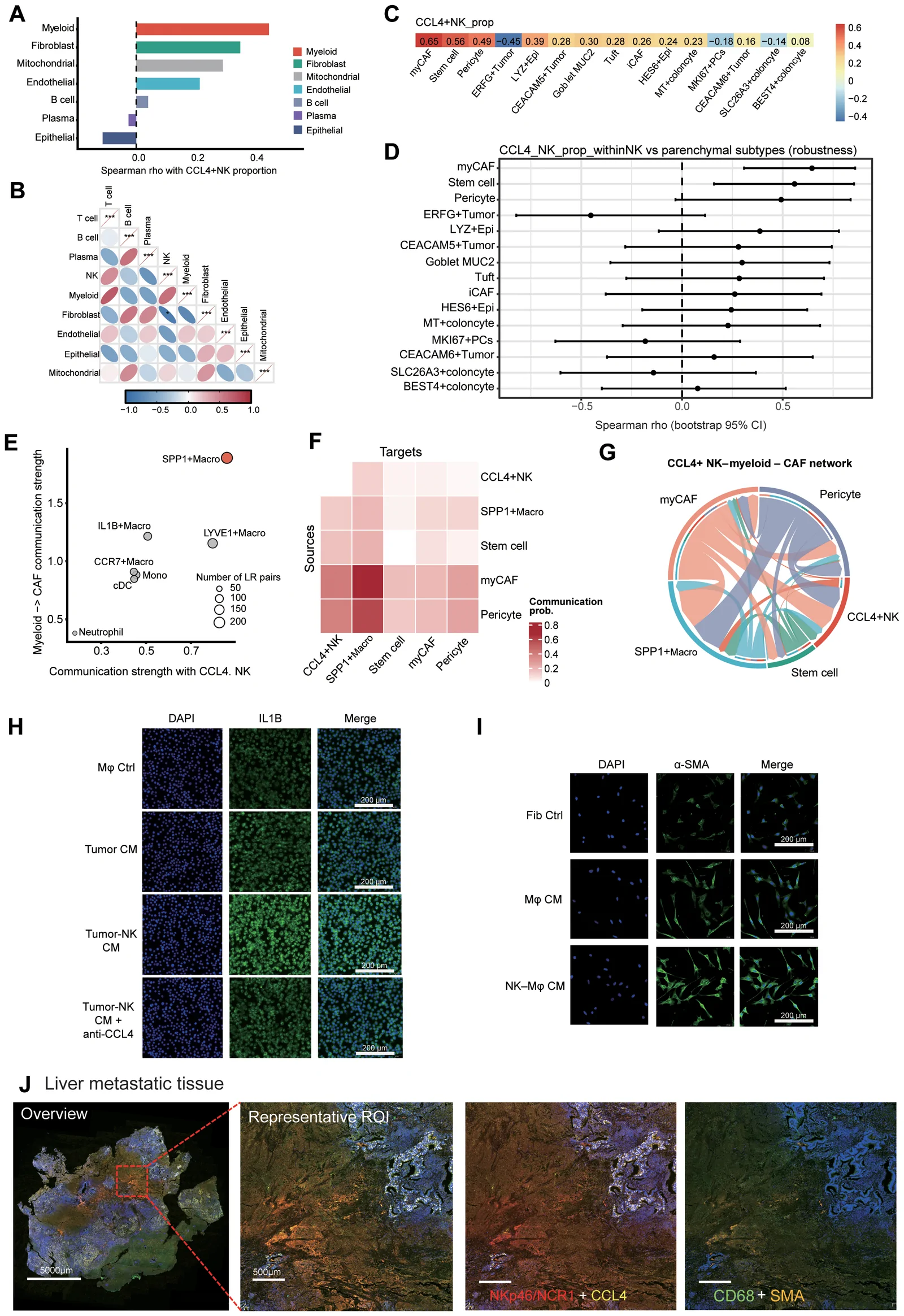
CCL4+ NK cells associate with an SPP1+ macrophage and stromal remodeling network. **(A)** Spearman correlations between the CCL4+ NK-cell proportion and major lineage proportions across biological samples. **(B)** Correlation matrix across major cell compartments. **(C)** Correlations between the CCL4+ NK-cell proportion and refined parenchymal or stromal states. Values indicate correlation coefficients. **(D)** Bootstrap robustness analysis after normalization within the NK compartment; points indicate median Spearman rho and bars indicate 95% confidence intervals. **(E)** Myeloid-to-CAF communication strength plotted against communication strength with CCL4+ NK cells. Circle size denotes the number of inferred ligand-receptor pairs. **(F)** Communication probability matrix among CCL4+ NK cells, SPP1+ macrophages, stem-like epithelial cells, myCAFs and pericytes. **(G)** Chord diagram summarizing the inferred NK, myeloid and stromal communication network. Inferred communication indicates signaling potential and not direct causality. **(H)** Macrophage IL-1β immunofluorescence under control, Tumor-CM, Tumor-NK CM, and Tumor-NK CM + anti-CCL4 conditions. Scale bars, 200 μm. **(I)** Alpha-smooth muscle actin (α-SMA) immunofluorescence in fibroblasts under control, macrophage-conditioned-medium, and NK–macrophage-conditioned-medium conditions. Nuclei were counterstained with DAPI. Scale bars, 200 μm. **(J)** Liver-metastasis mIF showing representative NKp46/NCR1, CCL4, CD68, and α-SMA signals. Overview and representative ROI images are shown; scale bars are indicated in the panels.

Consistently, ssGSEA scoring in TCGA COAD/READ bulk tumours showed positive correlations between CCL4+ NK, SPP1+ macrophage, pericyte and myCAF signatures, including CCL4+ NK with SPP1+ macrophage and pericyte scores, and SPP1+ macrophage with pericyte and myCAF scores, supporting a bulk-level NK–myeloid–stromal association (**Supplementary Figures 7E, F**, **8C**). We next examined whether these compositional associations were mirrored by predicted ligand-receptor communication. Ligand-receptor inference prioritized SPP1+ macrophages as the myeloid subset with the strongest predicted interaction with CCL4+ NK cells and outgoing signaling toward CAFs (**Figure 6E**). Communication matrices and network visualization represented CCL4+ NK cells, SPP1+ macrophages, myCAFs, pericytes and stem-like epithelial cells within a connected NK-myeloid-stromal circuit (**Figures 6F, G**, **Supplementary Figures 7D**, **8A, B**). Representative predicted ligand-receptor programs included inflammatory, chemotactic and matrix-remodeling signals, such as TGFB1, IFNG, MIF, SPP1, IL1B, COL1A1, FN1, THBS1 and PDGFB-linked interactions (**Supplementary Figures 6C–E**, **7C, D**, **8D, E**, **Supplementary Table 4**). These analyses indicated signaling potential, rather than direct ligand-receptor causality.

To functionally assess selected steps in the inferred NK–myeloid–stromal network, we established a conditioned-medium cascade. Tumor-conditioned NK-cell medium increased IL-1β signal in macrophages, whereas anti-CCL4 partially reduced this response; Tumor-CM alone also induced IL-1β, indicating both direct tumour-derived and NK-conditioned contributions (**Figure 6H**). In a downstream assay, macrophage-conditioned medium induced α-SMA expression in fibroblasts, and NK–macrophage conditioned medium further enhanced this myofibroblastic response (**Figure 6I**, **Supplementary Figure 10E**). Spatially, CCL4+ NK-cell signals in liver metastases were located closer to CD68-high macrophages than to α-SMA-high stromal cells, supporting a local NK–macrophage–stromal arrangement (**Figure 6J**). In the analyzed liver-metastasis section, the median nearest-neighbor distance from NKp46-high cells to CD68-high cells was 47.35 μm (IQR, 19.45–84.38 μm), compared with 75.62 μm (IQR, 23.82–159.84 μm) to α-SMA-high cells; this analysis is descriptive and does not establish direct contact or signaling. Together, these association, communication, spatial and *in vitro* data link CCL4+ NK cells to an SPP1+ macrophage–myCAF/pericyte remodelling network, while direct causality will require further perturbation studies.

### CCL4+ NK profiles co-localize with myeloid and stromal signatures

To assess cross-species overlap of the inflammatory and stromal programs associated with CCL4+ NK cells outside the human discovery cohort, we analyzed a murine CRLM dataset comprising 28,744 cells. These cells, sourced from primary tumors and liver metastases, were classified into major biological lineages encompassing epithelial/malignant populations, distinct immune subsets (neutrophils, macrophages, T/NK, and B/plasma cells), and stromal compartments (**Figures 7A, B**). Quality control metrics and canonical marker expression supported the fidelity of these murine celltype assignments (**Supplementary Figures 9A–E**).

**Figure 7:**
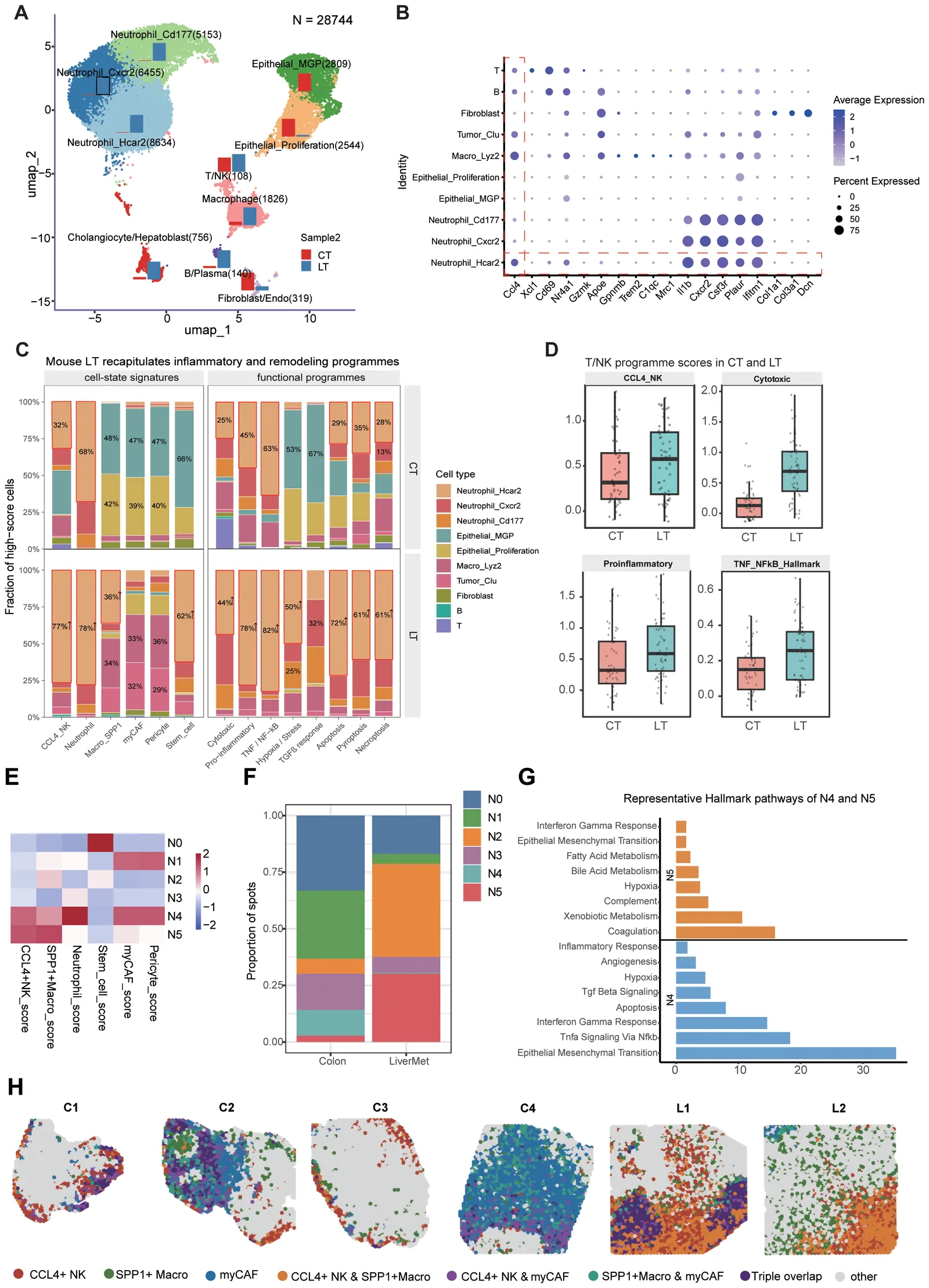
Mouse and spatial analyses provide exploratory cross-species program overlap and niche localization. **(A)** UMAP of mouse CRLM scRNA-seq data from CT and LT samples, showing annotated cell populations and retained cell numbers. The mouse comparison cohort comprised one CT sample and three LT samples. **(B)** Dot plot of representative marker genes across mouse cell types. Dot size denotes the percentage of expressing cells and colour denotes scaled mean expression. **(C)** Cross species scoring of human derived cell state signatures and functional programs in the mouse CRLM dataset after ortholog mapping. Stacked bars show the cell type composition of high scoring cells in CT and LT samples. **(D)** Descriptive comparison of CCL4+ NK, cytotoxic, proinflammatory and TNF/NF-κB Hallmark scores in mouse T/NK cells from CT and LT samples; this one-CT/three-LT comparison is exploratory. **(E)** Heatmap of scaled scRNA-seq derived signature scores across spatial niches N0–N5. **(F)** Proportions of spatial spots assigned to niches N0–N5 in colon and liver-metastatic sections. **(G)** Hallmark pathways enriched in N4 and N5 niches. **(H)** Spatial maps showing CCL4+ NK-, SPP1+ macrophage-, and myCAF-high regions and their pairwise and triple overlaps across colorectal primary-tumor sections C1–C4 and liver-metastatic sections L1–L2.

Gene signatures identified in the human discovery cohort were subsequently mapped to murine orthologs and scored across the dataset. Cells with high scores for the CCL4+ NK, neutrophil, SPP1+ macrophage, myCAF, pericyte and stem-like signatures mapped to their respective immune, epithelial and stromal compartments in both mouse colon tumor (CT) and liver metastasis (LT) samples (**Figure 7C**, **Supplementary Figures 9F, G**). Among murine T and NK cells, LT samples showed directionally higher CCL4+ NK, cytotoxic, proinflammatory and TNF/NF-κB Hallmark scores than CT samples (**Figure 7D**, **Supplementary Figure 9H**). Given the one-CT/three-LT biological-sample structure, this comparison is exploratory and supports cross-species program overlap rather than conservation of a defined lineage.

We next evaluated the spatial organization of these CCL4+ NK cell programs alongside myeloid and stromal remodeling signatures in human tissues. Spatial transcriptomic spots were stratified into six distinct niches (N0 to N5) based on signature scores derived from the single-cell reference (**Figure 7E**, **Supplementary Figure 10A**). Niches N4 and N5 were enriched for CCL4+ NK, SPP1+ macrophage, neutrophil, stem-like epithelial, myCAF, and pericyte signatures. N5 occupied a larger fraction of spots in liver metastases than in primary colorectal tumors (**Figures 7E, F**). Pathway analysis showed N4 enrichment for inflammation and tissue remodeling, encompassing epithelial-mesenchymal transition, TNF signaling via NF-κB, interferon-gamma response, apoptosis, TGF-β signaling, and hypoxia. In contrast, N5 exhibited prominent activation of metabolic and coagulation cascades, including xenobiotic, bile acid, and fatty acid metabolism (**Figure 7G**).

Spatial overlap analysis further showed enrichment of the CCL4+ NK program within macrophage and fibroblast rich regions. In subsets of primary colorectal tumor and liver metastatic sections, spots with high CCL4+ NK, SPP1+ macrophage and myCAF scores appeared in territories with pairwise or triple enrichment, whereas other sections showed more spatially segregated programs (**Figure 7H**, **Supplementary Figures 10B, C**). Ligand-receptor scoring in N4/N5 also enriched interactions involving TGFB1-TGFBR, SPP1-ITGAV/ITGB1, MIF-CD74/CXCR4, IFNG-IFNGR and COL1A1-CD44 (**Supplementary Figure 10D**). These spatial data are consistent with enrichment of the CCL4+ NK signature within inflammatory and fibroinflammatory metastatic niches, while higher resolution spatial profiling will be required to resolve direct cellular apposition among NK cells, macrophages and stromal cells. Representative multiplex immunofluorescence of liver metastatic tissue further showed NKp46/NCR1+ CCL4+ signal in regions containing CD68+ macrophage-enriched and α-SMA+ stromal areas (**Supplementary Figures 10F, G**).

### The IL-1β/NF-κB/CCL4 program is prioritized as a candidate inflammatory program

To explore regulatory programs associated with CCL4 within this inflammatory state, we performed a virtual knockout of the CCL4 gene using scTenifoldKnk. Downstream genes responsive to this perturbation mapped to pathways involved in stress and interferon responses, immune recognition, cytoskeletal regulation, vesicle trafficking, and autophagy. Notable responsive targets included KLRK1, IFITM1, RNASEK, HSPA1B, S100A6, SEPTIN7, and ATP6V0C (**Figure 8A**, **Supplementary Table 5**). Gene set enrichment linked the perturbed program to NK cell chemotaxis, lymphocyte chemotaxis, keratinocyte and epidermal differentiation, and viral life cycle processes (**Figure 8B**). These in silico data are consistent with CCL4 acting as a computationally inferred component of the inflammatory NK program.

**Figure 8:**
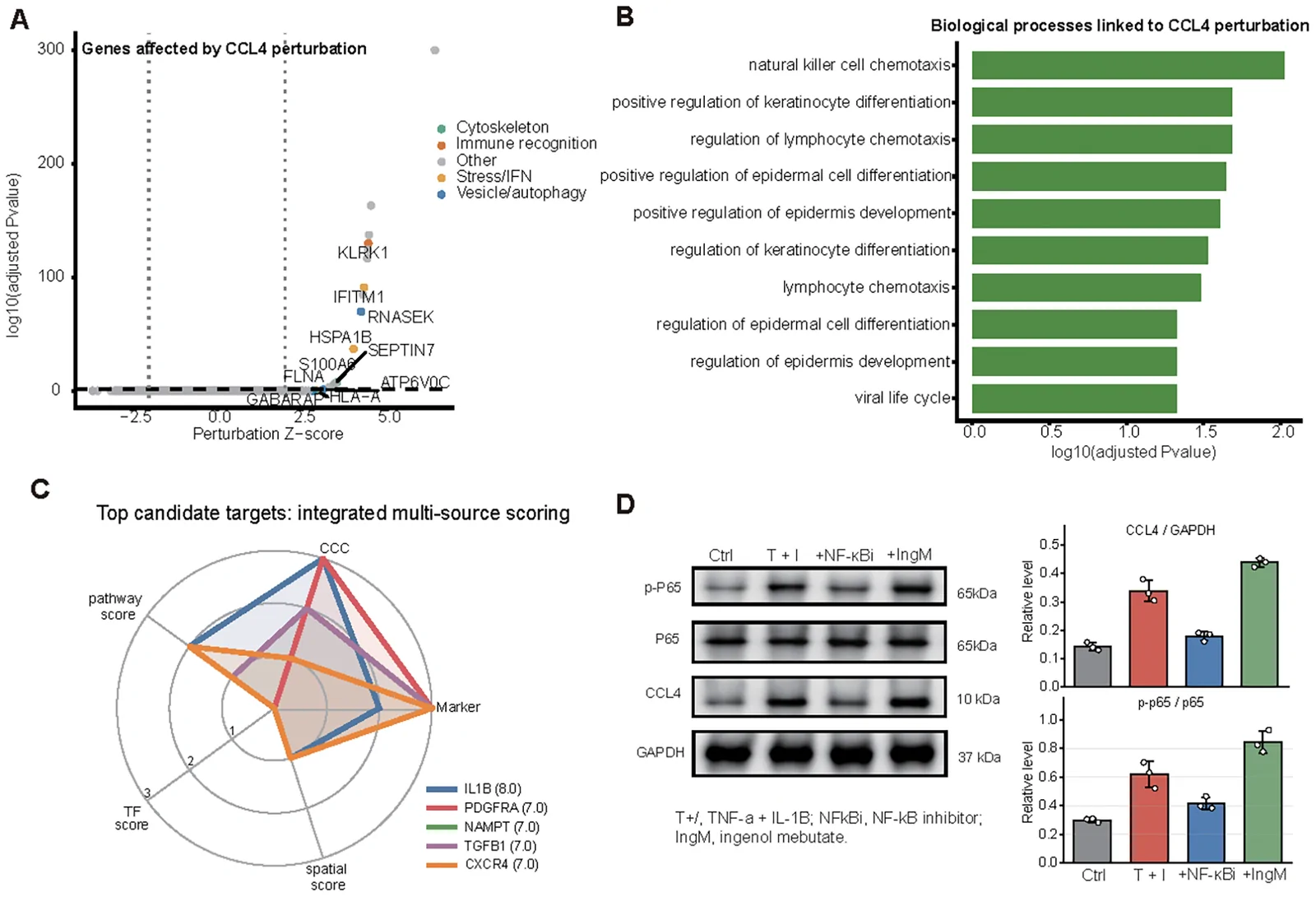
Virtual perturbation and NK92 immunoblotting provide convergent evidence for a candidate IL1B/NF-κB/CCL4 inflammatory program. **(A)** scTenifoldKnk virtual CCL4 perturbation in CCL4+ NK cells. Genes are displayed according to perturbation Z-score and −log10-adjusted P value, with selected genes grouped into cytoskeletal, immune-recognition, stress/interferon, vesicle/autophagy, and other functional categories. **(B)** Biological processes enriched among genes affected by virtual CCL4 perturbation. **(C)** Integrated target scoring based on marker expression, inferred communication, pathway activation, transcription factor activity and spatial support. Top ranked candidate nodes included IL1B, PDGFRA, NAMPT, TGFB1 and CXCR4. **(D)** Immunoblot analysis of p-p65, total p65, CCL4, and GAPDH in NK92 cells under the treatment conditions indicated in the panel: control, TNF-α plus IL-1β (T+I), NF-κB inhibitor (NF-κBi), and ingenol mebutate (IngM). Densitometric quantification of CCL4/GAPDH and p-p65/p65 was obtained from three independent experiments and is presented as mean ± SEM where applicable.

To prioritize candidate nodes within this inflammatory network, we integrated marker expression, ligand receptor inference, pathway activation, transcription factor activity and spatial localization. IL1B ranked as the top candidate node, followed by PDGFRA, NAMPT, TGFB1 and CXCR4 (**Figure 8C**, **Supplementary Table 8**). This multi-layer ranking prioritizes candidate inflammatory and stromal nodes for experimental follow up.

As an exploratory pharmacological layer, DrugReflector and scFOCAL were used to compare the CCL4+ NK transcriptional state with compound perturbation profiles. An ingenol-related profile annotated with PKC activation was retained for exploratory analysis (**Supplementary Figures 11A–D**, **Supplementary Tables 6**, **7**). We then ran a structure-based check for ingenol mebutate (67) in an IL-1β context. Redocking of the co-crystallized ligand T9C in the 8C3U IL-1β pocket gave an RMSD of 0.799 Å, and GNINA rescoring of a 1I1B IL-1β–ingenol mebutate pose yielded an affinity of -6.06 kcal/mol and a CNNscore of 0.7523 (**Supplementary Figures 11E, F**, **Supplementary Tables 9**, **10**). These analyses were used only for exploratory prioritization and do not establish target engagement or therapeutic activity.

Finally, we assessed pathway engagement at the protein level in NK92 cells. TNF-α plus IL-1β stimulation increased p-p65/p65 and CCL4/GAPDH levels relative to control cells; an NF-κB inhibitor reduced this response. Ingenol mebutate was included as an exploratory perturbation and increased p-p65/p65 and CCL4/GAPDH under inflammatory stimulation (**Figure 8D**). These computational and NK92 immunoblotting data provide exploratory support for an IL1B/NF-κB/CCL4 inflammatory program in CCL4+ NK cells and motivate future mechanistic studies.

## Discussion

Colorectal cancer liver metastasis (CRLM) remains difficult to treat because metastatic deposits develop within a specialized liver immune and stromal milieu (1–4, 68, 69). Single cell and spatial studies frame CRLM as an organized tissue ecosystem shaped by immune, myeloid, stromal and epithelial compartments rather than as a uniformly suppressed lesion (4–6, 35, 36, 70–73). In this context, our study identifies CCL4+ NK cells as an inflammatory NK-cell state associated with NF-κB signaling and enriched in primary colorectal tumors and liver metastases (**Figures 4**, **3**). The GSE164522 analysis independently reproduced this state in paired primary-tumor and liver-metastasis samples (**Supplementary Figure 4**). This finding broadens the interpretation of NK-cell remodeling in CRLM beyond relative compositional shifts and impaired cytotoxicity to include a chemokine-enriched program associated with inflammatory tissue remodeling.

These results refine current models of NK cell dysfunction in solid tumors. NK cells are commonly discussed through cytotoxic immune surveillance, exhaustion, impaired trafficking and adoptive cellular therapy (7, 9–12, 14, 15, 17, 74–76). Recent experimental data also show rapid functional impairment after tumor entry, pointing to the influence of local tissue cues (16). Our findings support a cell state model. NK cells showed lower relative representation within recovered T/NK cells in tumor-associated tissues and showed altered cytotoxic programs (**Figures 2E, F**), whereas the remaining NK compartment included CD56+ CCL4 and CD16+ CCL4 states with NF-κB, TNF, chemokine and stress-response programs (**Figures 4A–D**). In the independent GSE164522 cohort, the state was detected in paired samples from all 10 patients and showed a nominally higher CCL4-associated signal in liver metastases than in primary tumors (**Supplementary Figure 4**). Pseudotime, marker gene and transcription factor analyses positioned these cells along an inferred inflammatory remodeling continuum (**Figures 3B–H**, **Supplementary Figure 3**).

The clinical interpretation of this inflammatory NK state is context dependent. In bulk transcriptomic cohorts, CCL4 expression and CCL4-NK marker scores may capture broader host immune engagement, consistent with studies linking immune infiltration and immune contexture to outcome in colorectal cancer (18, 77, 78). Chemokines can recruit and organize immune cells, but their effects in cancer depend on the cellular source, receptor repertoire and tissue compartment (19, 79). In the present analysis, bulk-cohort analyses showed concordant inflammatory associations, whereas continuous Cox models did not support CCL4 expression or the CCL4-NK top-50 and top-100 signatures as stable prognostic variables independent of available clinical factors and total NK abundance (**Supplementary Figure 5**, **Supplementary Materials**). The CCL4-enriched state should therefore not be interpreted as uniformly protective or pathogenic, or as a stand-alone prognostic biomarker, on the basis of bulk associations alone. It marks an inflammatory NK program whose biological effect is shaped by the adjacent myeloid, stromal and epithelial compartments.

A key implication is that inflammatory NK states are integrated into myeloid and stromal remodeling niches. Sample-level analysis and ligand-receptor inference associated the CCL4-enriched state with SPP1-positive macrophage, myCAF and pericyte programs (**Figures 6A–G**, **Supplementary Figures 6**, **8**). This organization is consistent with prior work showing that tumor-associated myeloid cells and SPP1-positive macrophages regulate immune tone, extracellular matrix deposition and tissue architecture across solid tumors, including colorectal cancer (22–29, 31, 69, 80). It also aligns with the biology of cancer-associated fibroblasts, which generate matrix-dense tumor regions (30, 32–34, 81–83). In our data, predicted inflammatory and extracellular-matrix interactions connected CCL4-enriched NK, myeloid and stromal states (**Figures 6E–G**, **Supplementary Figure 8**). The conditioned-medium cascade provided *in vitro* support for selected steps of this association: a direct Tumor-CM control confirmed that SW620-derived factors affect macrophages, Tumor-NK CM produced a stronger IL-1β response, anti-CCL4 partially attenuated this signal, and downstream macrophage-conditioned medium increased fibroblast α-SMA (**Figures 6H, I**, **Supplementary Figure 10E**).

Mouse and spatial analyses supported this niche interpretation while defining its boundaries. The mouse CRLM dataset reproduced several human inflammatory and stromal programs at the signature level, showing cross-species program overlap rather than direct equivalence or conservation of a defined CCL4+ NK lineage (**Figures 7A–D**, **Supplementary Figure 9**). Spatial transcriptomics associated CCL4+ NK, SPP1+ macrophage, neutrophil, myCAF and pericyte signatures in remodeling niches, with N4 and N5 capturing inflammatory, stromal and metabolic tissue programs (**Figures 7E–H**, **Supplementary Figures 10A–D**). These findings are consistent with the concept that tumor immunity is organized into multicellular hubs rather than distributed uniformly throughout malignant tissue (35–39, 68, 69, 84). Multiplex immunofluorescence provided complementary tissue-level proximity evidence, showing local NKp46/NCR1-CCL4 signals in relation to CD68-high macrophage and α-SMA-high stromal regions (**Figure 6J**, **Supplementary Figure 10G**). Co-enrichment and proximity do not establish direct cellular contact, and higher-resolution lineage-resolved profiling will be required to test the proposed spatial relationships.

The perturbation analysis and supporting cell-model assays are consistent with an inflammatory IL1B/NF-κB/CCL4 program. Virtual CCL4 perturbation associated CCL4 with stress and interferon responses, immune recognition, cytoskeletal regulation and vesicle/autophagy-related programs, while integrated prioritization nominated IL1B, PDGFRA, NAMPT, TGFB1 and CXCR4 as candidate nodes (**Figures 8A–C**). IL-1 family cytokines regulate immune activation, myeloid polarization and stromal remodeling in a context-dependent manner (24, 80, 83, 85–87), while NF-κB connects inflammatory cytokine sensing to remodeling of the tumor microenvironment (88–90). Immunoblotting showed that TNF-α plus IL-1β increased NF-κB activation and the CCL4 protein response in NK92 cells, and that this response was reduced by NF-κB inhibition (**Figure 8D**). Ingenol mebutate was retained as an exploratory perturbation in this model system (67, **Supplementary Figure 11**); docking and transcriptional prioritization do not establish direct target engagement or therapeutic activity.

Several limitations should be considered. First, the discovery analysis used public single cell RNA sequencing datasets with limited sample numbers per tissue group, and the original cell-level comparisons may be influenced by tissue processing, sample composition and cell recovery. Second, communication inference estimates signaling potential from ligand and receptor expression; it does not demonstrate ligand-receptor causality or signaling direction *in vivo*. Third, pseudotime, virtual knockout and docking analyses are computational models and cannot substitute for direct perturbation experiments. Fourth, spatial transcriptomic spots contain multiple cells, and the archived-tissue mIF panels lacked CD45/CD3 lineage restriction. Fifth, the mouse and spatial datasets had limited biological replication. Finally, the *in vitro* cascade uses NK92, macrophage-like and fibroblast models, which simplify the cellular heterogeneity and spatial constraints of primary human liver metastases. Future studies should combine patient cohort validation, high-resolution spatial profiling, primary cell co-culture systems and *in vivo* perturbation.

Overall, this study characterizes a CCL4-enriched NK-cell transcriptional state associated with NF-κB signaling and embedded in myeloid and stromal remodeling niches in CRLM (**Figures 4**-**7**). NK-cell remodeling in metastatic colorectal cancer should therefore be interpreted through linked dimensions of altered cytotoxic programs, inflammatory activation and spatial niche organization. The NF-κB-CCL4-associated program provides a candidate framework for future mechanistic studies in patient-derived and *in vivo* models (**Figure 8**). Consistent with this framework, multiplex immunofluorescence showed local NKp46/NCR1-CCL4 co-occurrence and proximity to CD68-high and α-SMA-high tissue regions (**Figure 6J**, **Supplementary Figure 10G**).

## Data Availability

The datasets presented in this study can be found in online repositories. The names of the repository/repositories and accession number(s) can be found in the article/**Supplementary Material**.

## Supplementary material

Supplementary Figure 1 Quality control and integration of the discovery scRNA-seq cohort. **(a)** Numbers of cells retained in each sample before and after quality-control filtering. **(b)** Scatter plot showing nCount_RNA and nFeature_RNA, coloured by mitochondrial gene percentage. **(c)** Distributions of nFeature_RNA, mitochondrial percentage and hemoglobin gene percentage across samples. **(d)** UMAP visualization of annotated major cell compartments split by tissue group after data integration.

Supplementary Figure 2 Annotation and sample distribution of epithelial, stromal and B/plasma compartments. **(a)** UMAP visualization of epithelial and stromal cells coloured by individual sample. **(b)** UMAPs split by tissue group showing refined epithelial and stromal annotations, including tumor epithelial subsets, colonocyte subsets, goblet cells, tuft cells, stem-like epithelial cells, iCAFs, myCAFs and pericytes. **(c)** Dot plot of representative marker genes used for epithelial and stromal subset annotation. **(d)** UMAP visualization of B/plasma cells coloured by individual sample. (e) Dot plot of representative marker genes for B-cell and plasma-cell subsets. **(f)** Relative proportions of B-cell and plasma-cell subsets across tissue groups.

Supplementary Figure 3 Additional analyses supporting NK-cell functional remodeling and the CCL4+ NK transcriptional phenotype. **(a)** Volcano plots comparing LT and CT samples in all NK cells and in CCL4+ NK cells. Red and blue points indicate genes upregulated in LT and CT, respectively; grey points indicate non-significant genes. Representative genes are labelled. **(b)** Pathway-gene networks for CD56+ CCL4 and CD16+ CCL4 NK states, showing apoptosis, TNF signaling, NF-κB signaling and related pathways. Nodes represent genes or pathways, and edges indicate gene–pathway membership. **(c)** NF-κB PROGENy weights plotted against LT-CT mean expression differences. Representative genes are labeled. **(d)** CytoTRACE-based potency scores across NK-cell subclusters, showing comparable low differentiation potential among NK-cell states. **(e)** NK-cell pseudotime trajectory projected onto the UMAP embedding, illustrating the continuum of NK-cell state transitions. **(f)** Distribution of cytotoxic and chemokine/inflammatory scores in NK cells stratified by pseudotime quartiles. CCL4+ NK cells and other NK cells are shown separately to illustrate the shift between cytotoxic and inflammatory programs along pseudotime. **(g)** Correlation between apoptosis score and pseudotime in NK cells. The Pearson correlation coefficient and P value are shown. **(h)** Relative composition of pseudotime phases across samples. Phase 1, Phase 2 and Phase 3 represent early, intermediate inflammatory and late transition states, respectively.

Supplementary Figure 4 Independent GSE164522 patient-level and cluster-group validation of the CCL4-associated NK state. **(a)** Paired comparison of CCL4 top-100 score and the fraction of NK cells with detectable CCL4 transcripts between primary tumours (PT) and metastatic tumours (MT) in the GSE164522 cohort. Lines connect paired samples from the same patient. **(b)** Correlations between CCL4 top-100 score and proinflammatory or TNF/NF-κB scores across patient-condition pseudobulk samples. **(c)** Comparison of cytotoxic, TNF/NF-κB, proinflammatory and inhibitory/exhaustion-like program scores between cluster 0/1 and CCL4-associated cluster 2/3. **(d)** Representative multiplex immunofluorescence images showing CCL4+ NK cells and p-p65/NF-κB-IL-1β inflammatory signals in tumour tissues. Scale bars, 5000 μm for overview images and 100 μm for magnified images. P values and Benjamini–Hochberg-adjusted values are shown where applicable.

Supplementary Figure 5 Bulk-transcriptomic support for the CCL4+ NK–associated inflammatory remodeling program in colorectal cancer liver metastasis. **(a)** Volcano plot showing differential gene expression between liver metastasis (LM) and primary colorectal cancer samples in GSE50760. Selected genes associated with CCL4+ NK cells, SPP1+ macrophages, myCAFs, pericytes, and stem-cell states are highlighted. **(b)** CCL4 expression across liver metastasis, primary colorectal cancer, and normal colon samples in GSE50760. **(c)** Correlation between the CCL4+ NK single-sample gene set enrichment analysis (ssGSEA) score and the NF-κB ssGSEA score in GSE50760. The Spearman correlation coefficient and P value are indicated in the panel. **(d)** Exploratory Kaplan–Meier survival analyses stratified by CCL4+ NK marker scores in the combined TCGA-COAD/TCGA-READ cohort and in GSE159216. High- and low-score groups are shown as indicated in the panels. **(e)** ssGSEA score distributions for hypoxia/stress, SPP1+ macrophage, proinflammatory, and TNF/NF-κB programs across normal colon, primary colorectal cancer, and liver metastasis samples in GSE50760. Kruskal–Wallis P values are indicated in the panels. **(f)** Distribution of log2 fold changes for CCL4+ NK signature genes in GSE213402, grouped according to genes upregulated in colorectal cancer or liver metastasis. **(g)** Exploratory Kaplan–Meier survival analyses stratified by CCL4 expression in TCGA-READ and TCGA-COAD. Optimal cut-off values and log-rank P values are indicated in the panels.

Supplementary Figure 6 Pathway activation, inferred communication and tissue distribution of parenchymal and myeloid subsets. **(a)** PROGENy-inferred pathway activation across epithelial and stromal subtypes, showing pathway heterogeneity among epithelial, CAF and pericyte populations. **(b)** Feature plots of representative marker genes supporting myeloid subset annotation, including S100A8, SPP1, LYVE1, FCER1A, CCR7 and IL1B. **(c)** CellChat-inferred interaction weights between CCL4^+^ NK states and epithelial–stromal subsets in both epithelial/stromal-to-NK and NK-to-epithelial/stromal directions. **(d)** Representative ligand-receptor pairs linking CCL4+ NK states with myCAF, stem-like epithelial and pericyte states. **(e)** Recurrent CCL4+ NK-related communication axes ranked by the number of receiver cell types involved.

Supplementary Figure 7 Additional myeloid and CellChat analyses supporting the NK-myeloid-stromal network. **(a)** Differential number of inferred interactions between LT and CT across major cell compartments. **(b)** UMAP visualization of myeloid subclusters, including neutrophils, monocytes, dendritic cells and macrophage subsets. **(c)** Representative communication probabilities among CCL4+ NK, myeloid and CAF/stromal compartments. **(d)** Chord diagrams comparing inferred communication from CCL4+ NK, SPP1+ macrophage and neutrophil sender populations toward myCAF and pericyte receivers in CT and LT. **(e)** Relative composition of NK and myeloid subclusters between normal and tumor groups. **(f)** Correlations between CCL4+ NK, SPP1+ macrophage, pericyte and myCAF signature scores, supporting coordinated NK–myeloid–stromal remodelling.

Supplementary Figure 8 Correlation and ligand-activity analyses supporting the NK-myeloid-stromal remodeling network. **(a)** Kendall correlation heatmap showing pairwise associations among CCL4+ NK subsets, myeloid subsets, stromal cells and tumor epithelial subsets across samples. Asterisks indicate statistical significance. **(b)** Prioritised NK-myeloid-stromal communication network inferred by MultiNicheNet. Node colours denote cell populations, edge colours denote communication categories and edge width denotes prioritization score. **(c)** Sample-level correlations between CCL4+ NK subsets or SPP1+ macrophages and myCAF/pericyte proportions. **(d)** LT pericyte-focused ligand-receptor interactions prioritized from CCL4+ NK, SPP1+ macrophage and neutrophil senders, showing inflammatory, TGFβ-remodeling and ECM/vascular-remodeling inputs. **(e)** MultiNicheNet ligand-activity analysis showing prioritized ligands, inferred ligand activities, predicted target genes, regulatory potential and scaled pseudobulk expression in CT and LT samples.

Supplementary Figure 9 Mouse CRLM scRNA-seq analysis shows cross-species overlap in inflammatory and stromal-remodelling programs. **(a)** Representative images of the mouse CRLM model and harvested liver metastatic tissue. **(b)** Quality-control metrics for mouse scRNA-seq libraries, including nFeature_RNA and nCount_RNA across one CT sample and three LT samples. **(c)** UMAP embedding of the integrated mouse scRNA-seq dataset coloured by sample origin. **(d)** Relative composition of annotated mouse cell populations across individual samples and CT/LT groups. **(e)** Heatmap of canonical marker genes supporting mouse cell-type annotation. **(f)** Cross-species scoring of human-derived cell-state signatures in mouse cell types after ortholog mapping. **(g)** Module-score comparisons of representative remodeling-associated signatures between CT and LT samples. **(h)** LT versus CT differences in functional program scores, including apoptosis, cytotoxicity, hypoxia/stress, necroptosis, proinflammatory signaling, pyroptosis, TGFβ response and TNF/NF-κB programs.

Supplementary Figure 10 Spatial niche, ligand-receptor and cell-model analyses. **(a)** Spatial distribution of niches N0–N5 across colorectal primary-tumor sections C1–C4 and liver-metastatic sections L1–L2. **(b)** Spatial maps showing regions with high CCL4+ NK, SPP1+ macrophage, and pericyte signature scores, together with their pairwise and triple overlaps across C1–C4 and L1–L2. **(c)** Fractions of spatial spots assigned to individual or overlapping CCL4+ NK, SPP1+ macrophage, myCAF, and pericyte program categories. **(d)** Heatmap showing median log1p-transformed scores for selected ligand–receptor interactions across spatial niches N0–N5, including TGFB1–TGFBR, SPP1–integrin, MIF–CD74/CXCR4, IFNG–IFNGR, and COL1A1–CD44 signaling. **(e)** Cell-level distributions of IL-1β fluorescence intensity in macrophages under Mφ Ctrl, Tumor-NK CM, and Tumor-NK CM + anti-CCL4 conditions, and α-SMA fluorescence intensity in fibroblasts under Fib Ctrl, Mφ CM, and NK–Mφ CM conditions. Individual points represent segmented cells and are shown to visualize cell-level distributions; statistical inference was based on three independent biological experiments. **(f)** Representative hematoxylin and eosin (H&E)-stained tissue sections corresponding to colorectal primary-tumor and liver-metastatic specimens. **(g)** Multiplex immunofluorescence of liver-metastatic tissue showing an overview and representative ROI, together with NKp46/NCR1 and CCL4 signals and CD68 and α-SMA signals. Scale bars are indicated in the panels.

Supplementary Figure 11 Exploratory compound-prioritization and docking analyses. **(a)** DrugReflector Sankey plot showing candidate compounds, annotated mechanisms of action and primary targets. **(b)** scFOCAL volcano plot showing differential expression between predicted ingenol-mebutate-sensitive and -resistant states. Representative differentially expressed genes are labeled. **(c)** DrugReflector ranking of candidate compounds for the CCL4+ NK-associated transcriptional state. **(d)** scFOCAL ranking of compounds nearest to the ingenol mebutate perturbation profile based on combination score. **(e)** Redocking validation of the co-crystallized ligand T9C in the IL-1β binding pocket of Protein Data Bank structure 8C3U. The superimposed crystallographic and redocked poses are shown, with a root-mean-square deviation (RMSD) of 0.799 Å. **(f)** GNINA rescoring of the IL-1β-ingenol mebutate docking pose using the 1I1B IL-1β structure. The representative pose, predicted affinity and CNNscore are shown.
